# Integration of bioassay and non-target metabolite analysis of tomato reveals that β-carotene and lycopene activate the adiponectin signaling pathway, including AMPK phosphorylation

**DOI:** 10.1371/journal.pone.0267248

**Published:** 2022-07-01

**Authors:** Shinsuke Mohri, Haruya Takahashi, Maiko Sakai, Naoko Waki, Shingo Takahashi, Koichi Aizawa, Hiroyuki Suganuma, Takeshi Ara, Tatsuya Sugawara, Daisuke Shibata, Yasuki Matsumura, Tsuyoshi Goto, Teruo Kawada

**Affiliations:** 1 Laboratory of Molecular Function of Food, Graduate School of Agriculture, Kyoto University, Kyoto, Japan; 2 Laboratory of Technology of Marine Bioproducts, Graduate School of Agriculture, Kyoto University, Kyoto, Japan; 3 KAGOME Tomato Discoveries Laboratory, Graduate School of Agriculture, Kyoto University, Kyoto, Japan; 4 Innovation Division, KAGOME CO., LTD., Tochigi, Japan; 5 Kazusa DNA Research Institutes, Kazusa-Kamatari, Chiba, Japan; 6 Laboratory of Quality Analysis and Assessment, Graduate School of Agriculture, Kyoto University, Kyoto, Japan; 7 Research Unit for Physiological Chemistry, Kyoto University, Kyoto, Japan; Foshan University, CHINA

## Abstract

Adiponectin, an adipokine, regulates glucose metabolism and insulin sensitivity through the adiponectin receptor (AdipoR). In this study, we searched for metabolites that activate the adiponectin signaling pathway from tomato (*Solanum lycopersicu*). Metabolites of mature tomato were separated into 55 fractions by liquid chromatography, and then each fraction was examined using the phosphorylation assay of AMP-protein kinase (AMPK) in C2C12 myotubes and in AdipoR-knockdown cells by small interfering RNA (siRNA). Several fractions showed AMPK phosphorylation in C2C12 myotubes and siRNA-mediated abrogation of the effect. Non-targeted metabolite analysis revealed the presence of 721 diverse metabolites in tomato. By integrating the activity of fractions on AMPK phosphorylation and the 721 metabolites based on their retention times of liquid chromatography, we performed a comprehensive screen for metabolites that possess adiponectin-like activity. As the screening suggested that the active fractions contained four carotenoids, we further analyzed β-carotene and lycopene, the major carotenoids of food. They induced AMPK phosphorylation via the AdipoR, Ca^2+^/calmodulin-dependent protein kinase kinase and Ca^2+^ influx, in addition to activating glucose uptake via AdipoR in C2C12 myotubes. All these events were characteristic adiponectin actions. These results indicated that the food-derived carotenoids, β-carotene and lycopene, activate the adiponectin signaling pathway, including AMPK phosphorylation.

## Introduction

Adiponectin is an adipocyte-derived hormone that regulates glucose metabolism and insulin sensitivity by acting via an adiponectin receptor (AdipoR) [1]. The prevalence of obesity has markedly increased in recent years [2] and is generally associated with cardiovascular diseases, insulin resistance, and type-2 diabetes [3,4]. Previous studies have shown that the plasma levels of adiponectin are reduced in obesity [5], but replenishment of adiponectin levels can ameliorate glucose metabolism disorder [6]. In muscle and liver, adiponectin activates AMP-activated protein kinase (AMPK), thereby modulating glucose metabolism *in vitro* and *in vivo* [7]. Accordingly, screening for metabolites possessing adiponectin-like activities that target AMPK phosphorylation represents a valuable therapeutic approach for the treatment of glucose metabolism disorder in obesity.

Recently, a synthetic small-molecule adiponectin agonist, 2-(4-benzoylphnoxy)-N-[1-(phenylmethyl)-4-piperidinyl] acetamide (AdipoRon), was identified from a chemical library [8]. AdipoRon activated adiponectin signaling pathways, including AMPK phosphorylation, and contributed to the improvement of glucose metabolism disorder *in vivo*. In addition, several peptides have been reported to act as AdipoR agonists, activating the adiponectin signaling pathway and enhancing glucose uptake in muscle cells [9,10]. These reports indicate that low molecular weight compounds may activate the adiponectin signaling pathway and ameliorate glucose metabolism disorder. However, whether food ingredients contain small-molecules that activate adiponectin signaling pathway has not been fully investigated.

Tomato (*Solanum lycopersicum*) is highly valuable as a staple of the human diet and in terms of scientific significance. Novel techniques, such as whole genome sequencing and CRISPR editing, have been applied to tomato as a model system [11,12]. Tomato is a popular and extensively consumed crop, with many studies showing that tomato consumption is correlated with a reduced risk of chronic illnesses, including cancer, cardiovascular diseases, and type 2 diabetes [13–15]. Several active metabolites with the potential to ameliorate metabolic disorders have been identified from tomato [16–18], but the metabolites responsible for the aforementioned therapeutic benefits are not fully elucidated.

Non-targeted metabolite analysis including liquid chromatography coupled with ultraprecise mass spectrometry provide retention time and molecular weight data of comprehensive metabolites in samples. This allows for a high sensitivity and high-throughput comprehensive annotating of metabolites in information processing, and then lists of global estimated metabolites in samples (the metabolome) are created [19,20]. Therefore, non-targeted metabolite analysis has been increasingly used for screening for valuable metabolites in various research fields. In plant and food research, metabolomics helps to elucidate the quality, taste, yield, and stress tolerance of plants [21,22]. In the medical field, non-targeted metabolite analysis has been used to discover early diagnostic biomarkers of diseases [23,24]. In biochemistry field, metabolites exerting specific biological effects on stem cell differentiation [25], type-2 diabetes [26,27], and immune cell activation [28] have been identified from lists of global estimated metabolites annotated by non-targeted metabolite analysis.

Here we identified food-derived metabolites that activate adiponectin signaling pathway from tomato. Extract from mature tomato exhibited phosphorylation of AMPK in myotubes differentiated from C2C12 immortalized mouse myoblasts in a dose-dependent manner. This effect of tomato was abrogated in AdipoR-knockdown cells by small interfering RNA (siRNA). These results suggested that the tomato contained metabolites that activate adiponectin signaling pathway, including AMPK phosphorylation. To identify the molecules possessing adiponectin-like activity, tomato metabolites were screened by integrating the biological assay of high performance liquid chromatography (HPLC) fractions and non-targeted metabolite analysis based on their retention times. Since this screening suggested that several carotenoids have adiponectin-like activity, we selected β-carotene and lycopene, the major metabolites of the food, for further characterization. Our results showed that the carotenoids activated the adiponectin signaling pathway, including AMPK phosphorylation.

## Materials and methods

### Plant material and chemicals

The raw tomatoes were provided by KAGOME CO., LTD. (Nasushiobara, Tochigi, Japan; identifier no. *KTP001*). Unless otherwise specified, all chemicals used were from Invitrogen Corp. (Carlsbad, CA, USA), Nacalai Tesque Inc. (Kyoto, Japan), CaroteNature (Münsingen, Switzerland), or Wako (Osaka, Japan), and were guaranteed to be of reagent-, HPLC-, or tissue culture-grade.

### Extraction and fractionation

The extraction and fractionation of the tomatoes were performed as follows. The hydrophilic extract was obtained from a freeze-dried tomato by homogenization in a methanol/water mixture (8/2, v/v) at room temperature. The sample was centrifuged (15,000 rpm, 10 min, 4°C) and the supernatant was collected. A mixture of methanol/methyl tert-butyl ether (1/3, v/v) was subsequently added to the collected supernatant, centrifuged (10,000×*g*, 5 min, 4°C), and the lower layer was collected as the hydrophilic tomato extract. The hydrophobic extract was obtained from non-dried, frozen tomato using a mixture of methanol/methyl tert-butyl ether (1/3, v/v) homogenized at room temperature. After the addition of a methanol/water (1/3, v/v) mixture to the homogenized tomato, the sample was centrifuged (10,000×*g*, 5 min, 4°C), and the colored upper layer was collected as the hydrophobic tomato extract. The hydrophobic tomato extract was fractionated using reversed-phase HPLC on a C_30_-YMC carotenoid column (4.6×250 mm; YMC CO., LTD.) with solvents A (methanol/water, 95/5, v/v) and B (methyl tert-butyl ether/methanol, 70/30, v/v) as the mobile phase. The gradient program began with 100% solvent A for 2.0 min followed by a linear elution gradient from 100% to 0% of solvent A in solvent B for 25.0 min. Isocratic flow of 100% solvent B was maintained from 25.0 to 45.0 min. A linear gradient from 100% to 0% solvent B in solvent A for 0.1 min was then used. Subsequently, 100% solvent A was isocratically maintained from 45.1 to 55.0 min. A diode array detector was used to monitor the HPLC eluate from 200–700 nm. The flow rate was 0.8 mL/min and the eluates were collected at 0.8 mL/fraction. The solvents in extracts and eluted fractions were evaporated under vacuum at 37°C using a rotary evaporator. The effects of evaporated samples were re-dissolved. 9-*cis*- and 13-*cis*-lycopene were not commercially available and hence these isomers were prepared as previously reported [29,30]. Briefly, all-*trans* lycopene was dissolved in chloroform and heated at 50°C for 16 h. The isomerized lycopene solutions were subsequently evaporated using a rotary evaporator (This residue of the isomerized lycopene was used in animal experiments). The residue was separated and collected using reverse-phase HPLC on a C_30_-YMC carotenoid column (10×250 mm; YMC CO., LTD., Kyoto, Japan) using solvent A (100% methanol) and solvent B (100% methyl tert-butyl ether) as the mobile phase at 5.0 mL/min. The program began with 60% solvent B in solvent A followed by a linear elution gradient from 60 to 75% solvent B for 30 min. A linear gradient was from 75 to 60% solvent B was then employed for 0.01 min, and 60% solvent B was run isocratically from 30 to 40 min. The obtained isomers were analyzed for absorption maxima and identified according to the literature [30].

### Metabolomic analysis

LC-MS for metabolomic analysis was performed using an HPLC apparatus (Agilent) coupled to an LTQ Orbitrap XL-MS system (Thermo Fisher Scientific Inc., San Jose, CA, USA), equipped with an electrospray source in APCI positive-ion mode. The spray capillary temperature was 200°C. Scan event 1 was performed in full mass mode (analyzer; orbitrap), and scan events 2–5 were operated in MS/MS mode (analyzer; ion trap, act type; collision-induced dissociation, normalized collision energy: 35.0). The tomato extract was injected and analyzed by LC, using the same LC analysis program as that used for HPLC. The data were obtained using Xcalibur (Thermo Fisher Scientific Inc.), Compound Discoverer 2.1 (Thermo Fisher Scientific Inc.), and PowerGet software (Kazusa DNA Research Institute, Chiba, Japan) using previously described methods [31–33]. Compound Discoverer software and PowerGet software were linked to the metabolite databases (KEGG, ChemSpider, and PubChem).

### Cell culture

Mouse skeletal myoblasts C2C12 were cultured in high-glucose Dulbecco’s modified Eagle’s medium (DMEM, Nacalai Tesque Inc.) with 10% fetal bovine serum, 100 U/mL penicillin, and 100 μg/mL streptomycin, at 37°C under a humidified atmosphere with 5% CO_2_. For myotube differentiation, C2C12 myoblasts (2×10^5^ cells/mL) were seeded into 12-well plates in DMEM for 3 d. The medium was subsequently replaced with DMEM containing 2% horse serum, 100 U/mL penicillin, and 100 μg/mL streptomycin for 5 d. The medium was replaced with fresh medium every 2 d. To evaluate the phosphorylation of AMPK, ACC, and p38 or the translocation of Glut4 and LKB1 or glucose uptake, the C2C12 myotubes were incubated in serum-free DMEM overnight and subsequently treated in serum-free medium.

### RNA interference

Three days after the differentiation induction, cells were transiently transfected using the transfection reagent, Lipofectamine 2000 (Invitrogen). AdipoR1 (Mm_Adipor1_3, QIAGEN) and a non-targeted control siRNA (MISSION siRNA universal negative control, Sigma-Aldrich) were used. The siRNA molecules (final concentration: 60 pmol) and 5 μL of Lipofectamine were first diluted in 60 μL of reduced-serum medium (Opti-MEM; Invitrogen) and then mixed. The mixture was incubated for 20 min at room temperature and 120 μL of the mixture was added in a drop-wise fashion to each culture well containing 600 μL of serum-free DMEM. The cells were used in the ensuing experiments 48 h after transfection. The AdipoR1-knockdown of C2C12 myotubes by siRNA transfection was examined by immunoblotting (S1A Fig).

### Immunoblotting

Proteins from C2C12 myotubes were solubilized in lysis buffer (50 mM Tris-HCl, 5 mM EDTA, 150 mM NaCl, 0.5% Nonidet P-40, 1.0% protease inhibitor, and 1.0% phosphatase inhibitor cocktail; Nacalai Tesque). The protein concentration in the cell lysate was determined using a detergent-compatible protein assay (BioRad Laboratories, Hercules, CA, USA). For detecting the level of Glut4 translocation in the cell, the plasma membrane fraction was extracted using the Mem-PER Plus Membrane Protein Extraction Kit (Cat. 89842, Thermo Scientific), according to the manufacturer^’^s protocol. For measuring the level of LKB1 translocation, the cytoplasmic fraction was extracted using Nuclear/Cytosolic Fractionation Kit (Cat. AKR-171, Cell Biolabs), according to the manufacturer^’^s protocol. The protein samples were resolved by sodium dodecyl sulfate-polyacrylamide gel electrophoresis and transferred onto a polyvinylidene fluoride membrane (Millipore, Bedford, MA, USA). After blocking in 5.0% skim milk in TBS containing 0.1% Tween-20 for 1 h, the blots were incubated with the anti-AMPK (1:2000 dilution, Cat. # 5831, Cell Signaling), anti-phosphorylated AMPK (1:2000 dilution, Cat. # 2535, Cell Signaling), anti-ACC (1:1000 dilution, Cat. # 3662, Cell Signaling), anti-phosphorylated ACC (1:1000 dilution, Cat. # 3661, Cell Signaling), anti-p38 (1:1000 dilution, Cat. # 9212, Cell Signaling), anti-phosphorylated p38 (1:1000 dilution, Cat. # 9215, Cell Signaling), anti-AdipoR1 (1:100 dilution, Cat. sc-518030, SANTA CRUZ), anti-βActin (1:2000, Cat. # 4967, Cell Signaling), anti-LKB1 (1:500 dilution, Cat. # 3050, Cell Signaling) and anti-Glut4 (1:500 dilution, Cat. # 2213, Cell Signaling) antibodies overnight. A secondary antibody conjugated with horseradish peroxidase (Santa Cruz Biotechnology, Dallas, Texas, USA) was then added. The secondary antibody was visualized by incubation with a chemiluminescent horseradish peroxidase substrate (Millipore), and the protein bands were quantified using ImageJ (National Institutes of Health, Bethesda, MD, USA).

### 2-Deoxyglucose uptake

The uptake of 2-deoxyglucose by C2C12 myotubes was evaluated as follows. The cells (2×10^5^ cells/mL) were washed three times with warm HKR buffer (20 mM HEPES, pH 7.4, 140 mM NaCl, 5 mM KCl, 2.5 mM MgSO_4_, and 1 mM CaCl_2_). After 10 min at 37°C, the cells were incubated with 2-deoxy-d [H^3^] glucose (1 μCi/well) for 10 min at 37°C. The reaction was terminated by washing with ice-cold phosphate-buffered saline three times. The cells were lysed in 0.1 N NaOH (400 μL/well) and cell radioactivity was subsequently evaluated by scintillation counting (LS6500, Beckman Coulter, Inc., CA, USA). Glucose uptake values were corrected for non-carrier mediated transport by measuring glucose uptake in the presence of 10 μM cytochalasin B.

## Animal experiments

Five-week-old male *db/db* mice were purchased from CLEA Japan (Tokyo, Japan). Mice were kept in individual cages in a temperature-controlled room at 23 ± 1°C and maintained under a constant 12 h light/dark cycle. Mice had access to commercial chow diet (MF; Oriental Yeast Co. Ltd., Tokyo, Japan) and water *ad libitum* for 3 weeks. Eight-week-old mice were divided into different treatment groups (control, n = 6 per group; all-*trans* lycopene, n = 6 per group; *cis* lycopene, n = 7 per group). After fasting for 7.5 hours, all-*trans* lycopene (200mg/kg body weight) and *cis*-lycopene (200mg/kg body weight) were orally administered once and blood samples were collected. Plasma glucose level was determined with glucose CII-test Kit (Wako, Osaka, Japan). All the animal experiments were approved by the Animal Care Committee of Kyoto University.

## Statistical analysis

All data are presented as the mean ± SEM. The data were analyzed by Student’s *t*-test or one-way ANOVA and multiple comparison tests (Dunnett’s or Tukey–Kramer). Differences were considered significant at *p* < 0.05.

## Results

### Assessment of adiponectin-like activity of tomato extract

**Fig 1:**
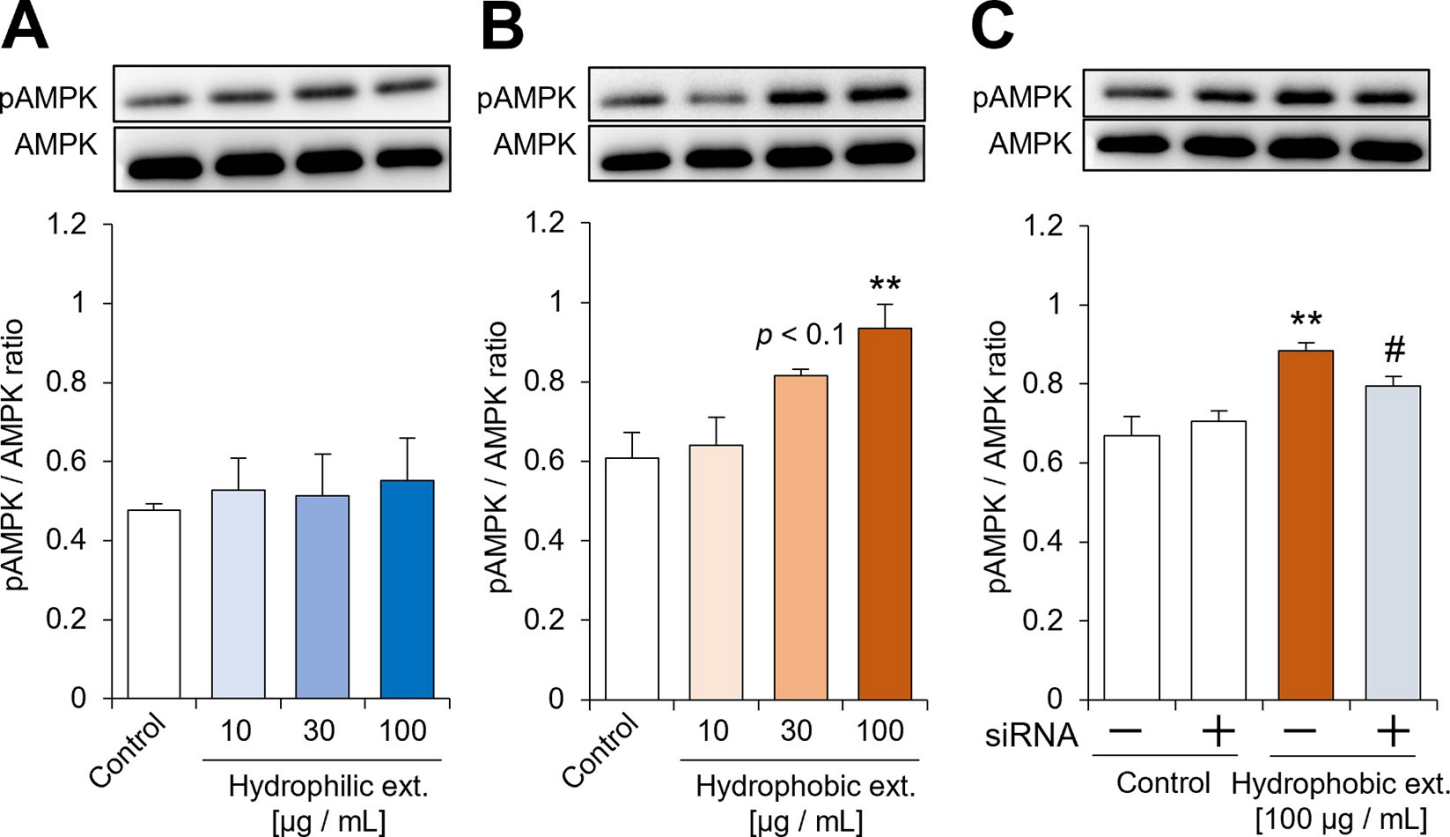
Assessment of adiponectin-like activity of tomato extract. **(A)** Effect of the hydrophilic tomato extract on AMPK phosphorylation in C2C12 myotubes. **(B)** Effect of the hydrophobic tomato extract on AMPK phosphorylation in C2C12 myotubes. **(C)** Effect of the hydrophobic tomato extract on AMPK phosphorylation in the presence and absence of AdipoR siRNA. In **(A)–(C)**, C2C12 myotubes were incubated with the extract for 10 min, then total protein was extracted from cell lysates of the treated C2C12 myotubes and analyzed by western blotting. Data are presented as mean ± standard error of the mean (SEM) from independent experiments (n = 4–5/group). **p < 0.01 vs. control. ^**#**^*p* < 0.05 vs. hydrophobic extract alone. pAMPK, phosphorylated AMPK; AMPK, total AMPK; ext, extract; siRNA, AdipoR siRNA.

For preliminary selection of the extract containing metabolites that activate adiponectin signaling pathway, hydrophilic and hydrophobic tomato extracts were prepared. A biological assay was used to evaluate the ability of the two tomato extracts to phosphorylate AMPK in C2C12 myotubes. The hydrophilic extract did not significantly affect AMPK phosphorylation (Fig 1A), whereas the hydrophobic extract caused an increase in AMPK phosphorylation similar to adiponectin, in a dose-dependent manner (Fig 1B). Furthermore, to confirm the dependency on the AdipoR, we investigated the effect of siRNA knockdown of AdipoR on AMPK phosphorylation induced by the hydrophobic tomato extract. Treatment with AdipoR siRNA inhibited the enhancement of AMPK phosphorylation by the hydrophobic extract (Fig 1C), suggesting that the hydrophobic tomato extract contained active metabolites possessing adiponectin-like activity on AMPK phosphorylation.

### Assessment of adiponectin-like activity of HPLC fractions of tomato

**Fig 2:**
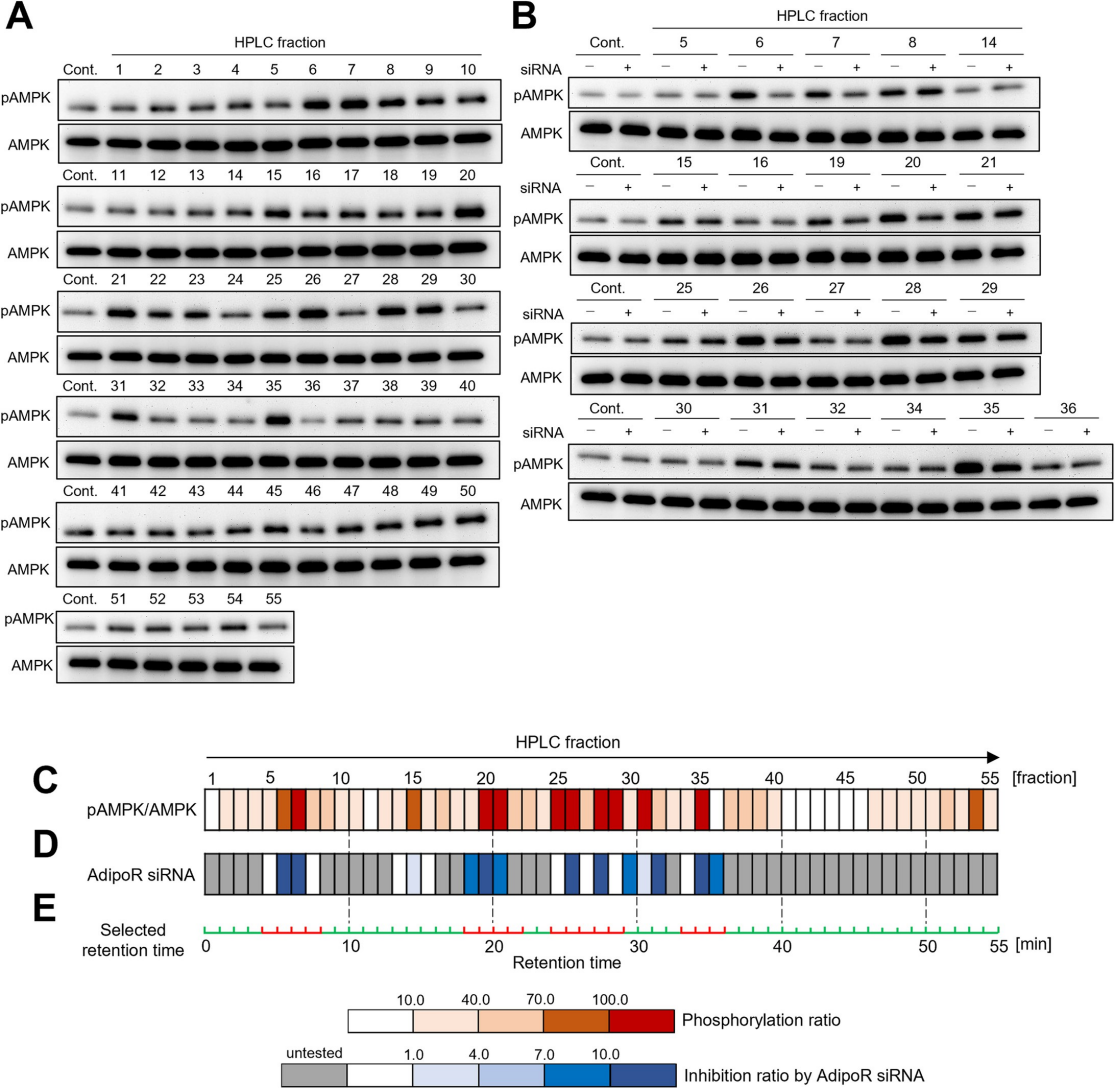
Assessment of the adiponectin-like activity of tomato-derived fractions. **(A)** Effect of the HPLC fractions of the hydrophobic tomato extract on AMPK phosphorylation in C2C12 myotubes. The HPLC fractions (no. 1–55) were separated using the same analytical conditions as those typically used for metabolomics (see details in the Methods section). **(B)** Effect of the selected HPLC fractions on AMPK phosphorylation in C2C12 myotubes in the absence and presence of AdipoR siRNA. **(C)** Heatmap of the AMPK phosphorylation ratio from Fig 2A. The phosphorylation ratios were calculated using the following equation: [(ratio for treated sample/ratio for control sample) × 100] –100. AMPK phosphorylation was normalized to the amount of AMPK. **(D)** Heatmap of the inhibition ratio of AMPK phosphorylation in Fig 2B. The inhibition ratio by AdipoR siRNA was calculated using the following equation: 100 –[(ratio for cells transfected with AdipoR siRNA/ratio for mock-transfected cells) × 100]. AMPK phosphorylation was normalized to the amount of AMPK. **(E)** Selected retention times (shown in red) for the primary target ranges of the fractions containing metabolites that activate adiponectin signaling pathway. Cont, control; pAMPK, phosphorylated AMPK; AMPK, total AMPK.

To select the primary targets of metabolites that activate adiponectin signaling pathway, we assessed the adiponectin-like activity of HPLC fractions of the hydrophobic tomato extract. The tomato extract was separated into 55 fractions. Functional assays were subsequently performed to determine the ability of these HPLC fractions to enhance the phosphorylation of AMPK, similar to adiponectin. A few fractions enhanced the phosphorylation of AMPK (Fig 2A and 2C). Henceforth, we focused on 21 fractions (no. 5–8, 14–16, 19–21, 25–32 and 34–36) centred on eight highly active fractions (no. 6, 7, 15, 20, 26, 28, 31 and 35). Next, the dependency of AMPK phosphorylating activity of these 21 fractions on AdipoR was assessed. We tested the effect of AdipoR siRNA on the phosphorylation of AMPK elicited by the 21 fractions. This assay revealed that AdipoR siRNA treatment inhibited AMPK phosphorylation by several fractions (no. 6, 7, 19, 20, 21, 26, 28, 30, 32, 35 and 36) (Fig 2B and 2D). The combination of increased phosphorylation ratio and increased inhibition ratio by AdipoR siRNA led us to focus on the four meaningful retention time ranges (red range in Fig 2E) near seven principal fractions (no. 6, 7, 20, 21, 26, 28 and 35) as primary targets containing metabolites that activate adiponectin signaling pathway.

### Screening for metabolites possessing adiponectin-like activity from tomato by integrating biological assay and non-targeted metabolite analysis

**Fig 3:**
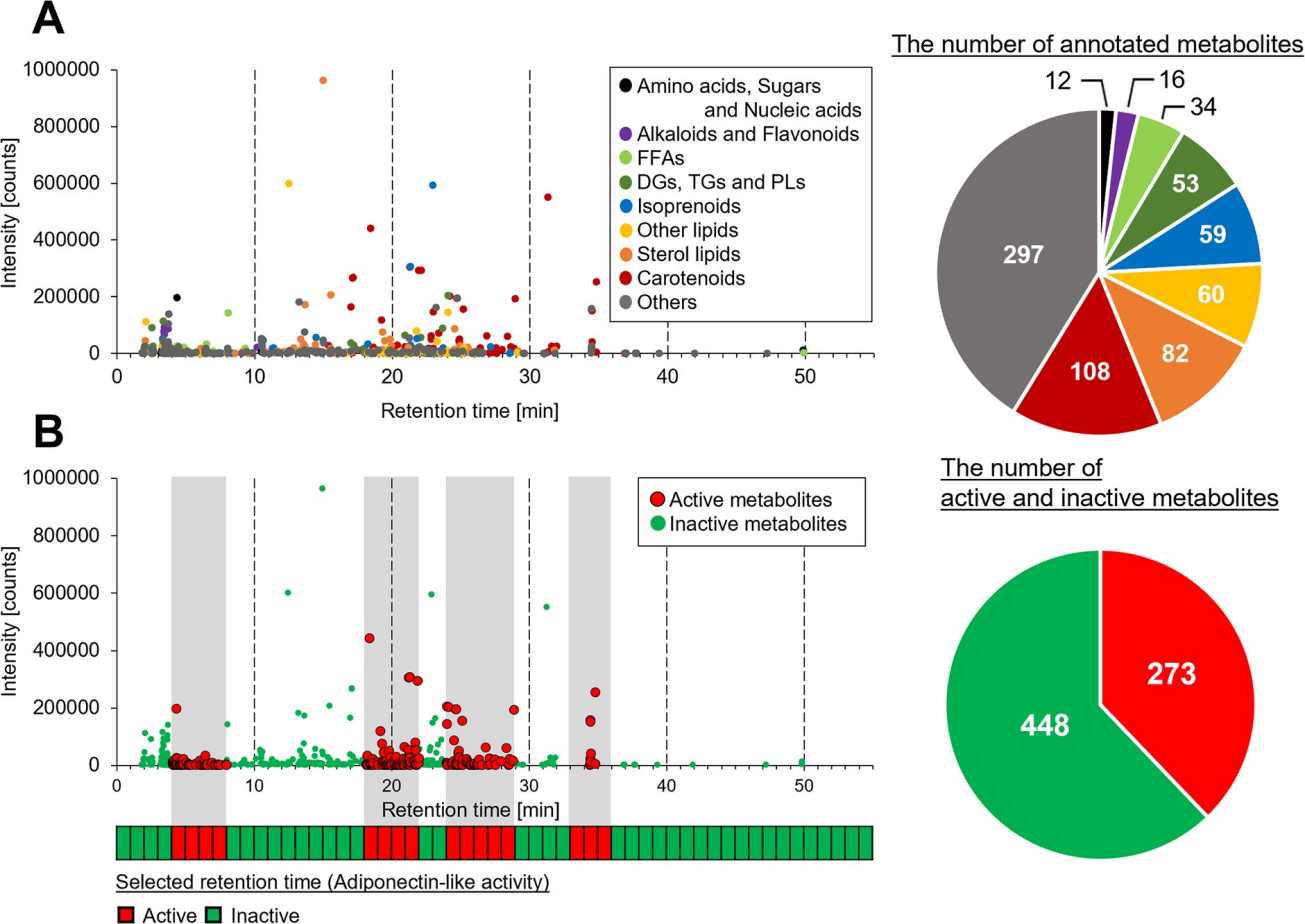
Screening for metabolites possessing adiponectin-like activity by the integration of the biological assay and non-targeted metabolite analysis. **(A)** Metabolome of the hydrophobic tomato extract. Left: Plot of the intensity and retention time of the annotated metabolites in the hydrophobic tomato extract. Each dot colour indicates the metabolite category. Right: The number of annotated metabolites in the hydrophobic tomato extract. Each colour indicates the metabolite category (For further details of annotated metabolites, see S1 Table). **(B)** Screening of the tomato metabolome targeting adiponectin-like activity by the integrated approach. Top: Plot of the intensity and retention time of the tomato metabolites. Grey zones correspond to retention times with adiponectin-like activity, and the red and green dots indicate active and inactive metabolites, respectively. Bottom: The range of retention times exhibiting adiponectin-like activity. The heatmap shows the active (red) or inactive (green) retention time ranges. Right: The number of screened active and inactive metabolites.

To screen for metabolites that activate adiponectin signaling pathway from tomato, we integrated the biological assay and non-targeted metabolite analysis. A comprehensive estimated metabolites in the hydrophobic tomato extract was annotated using non-targeted metabolite analysis under the same analytical conditions as those used for the HPLC fractionation (column type, mobile phase gradient, etc.). The tomato extract contained a wide variety of 721 metabolites, such as carotenoids, lipids, isoprenoids, flavonoids, amino acids and other compounds (Fig 3A). Integration of this metabolomic data and retention time ranges of the HPLC fractions that exhibited adiponectin-like activity revealed the presence of 273 metabolites that could potentially activate adiponectin signaling pathway from a diverse mixture of 721 metabolites (Fig 3B).

**Fig 4:**
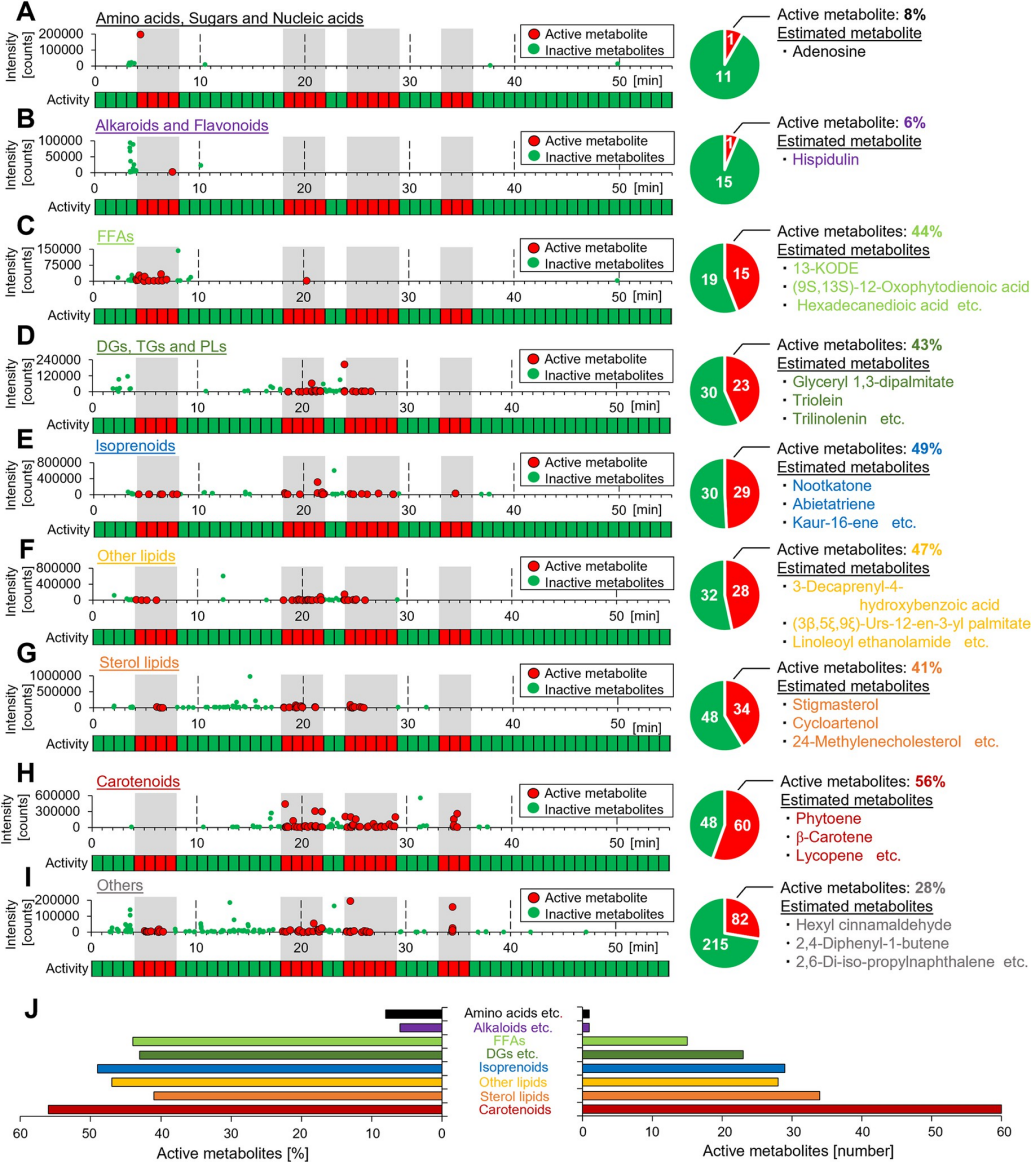
Screening for metabolites possessing adiponectin-like activity by the integration of the biological assay and non-targeted metabolite analysis in each structural category. **(A)–(I)** Left: Integration of the biological assay and non-targeted metabolite analysis in each structural category; **(A)**, amino acids, sugars and nucleic acids; **(B)**, alkaloids and flavonoids; **(C)**, free fatty acids (FFAs); **(D)**, diacylglycerols (DGs), triacylglycerols (TGs) and phospholipids (PLs); **(E)**, isoprenoids; **(F)**, other lipids; **(G)**, sterol lipids; **(H)**, carotenoids and **(I)**, others. Right: The number of screened active and inactive metabolites. The proportion of active metabolites and representative estimated metabolites annotated by non-targeted metabolite analysis are provided (see S1 Table for further details). **(J)** The proportion and number of active metabolites for each structure category.

In the next screening step, we integrated the biological assay and the non-targeted metabolite analysis to investigate 721 metabolites in each structural category. We detected 12 metabolites in the amino acids/sugars/nucleic acids category, one of which (8%) was active and annotated as adenosine (Fig 4A). Of the 16 detected alkaloids/flavonoids, one (6%) was active and annotated as hispidulin (Fig 4B). Of the 34 detected free fatty acids (FFAs), 15 (44%) were active and annotated as 13-KODE, (9S,13S)-12-oxophytodienoic acid, hexadecanedioic acid, etc. (Fig 4C). Of the 53 detected diacylglycerols, triacylglycerols, and phospholipids (DGs/TGs/PLs), 23 (43%) were active and annotated as glyceryl-1,3-dipalmitate, triolein, trilinolenin, etc. (Fig 4D). Of the 59 detected isoprenoids, 29 (49%) were active and annotated as nootkatone, abietatriene, kaur-16-ene, etc. (Fig 4E). Of the 60 detected other lipids, 28 (47%) were active and annotated as 3-decaprenyl-4-hydroxybenzoic acid, (3β,5ξ,9ξ)-Urs-12-en-3-yl palmitate, linoleoyl ethanolamide, etc. (Fig 4F). Of the 82 detected sterol lipids, 34 (41%) were active and annotated as stigmasterol, cycloartenol, 24-Methylenecholesterol etc. (Fig 4G). Of the 108 detected carotenoids, 60 (56%, the highest proportion of all categories) were active and annotated as phytoene, β-carotene, lycopene, etc. (Fig 4H). Of the 297 detected “other metabolites,” 82 (28%) were active and annotated as hexyl cinnamaldehyde, 2,4-diphenyl-1-butene, 2,6-di-iso-propylnaphthalene, etc. (Fig 4I). These activity-based metabolite profiling by integrating the biological assay and the non-targeted metabolite analysis suggested that a wide variety of tomato metabolites could potentially activate adiponectin signaling pathway. Furthermore, this screening revealed that carotenoids were the most meaningful active metabolites of tomato in terms of the adiponectin-like activity (Fig 4J).

### Identification of metabolites that activate adiponectin signaling pathway from tomato carotenoids

**Fig 5:**
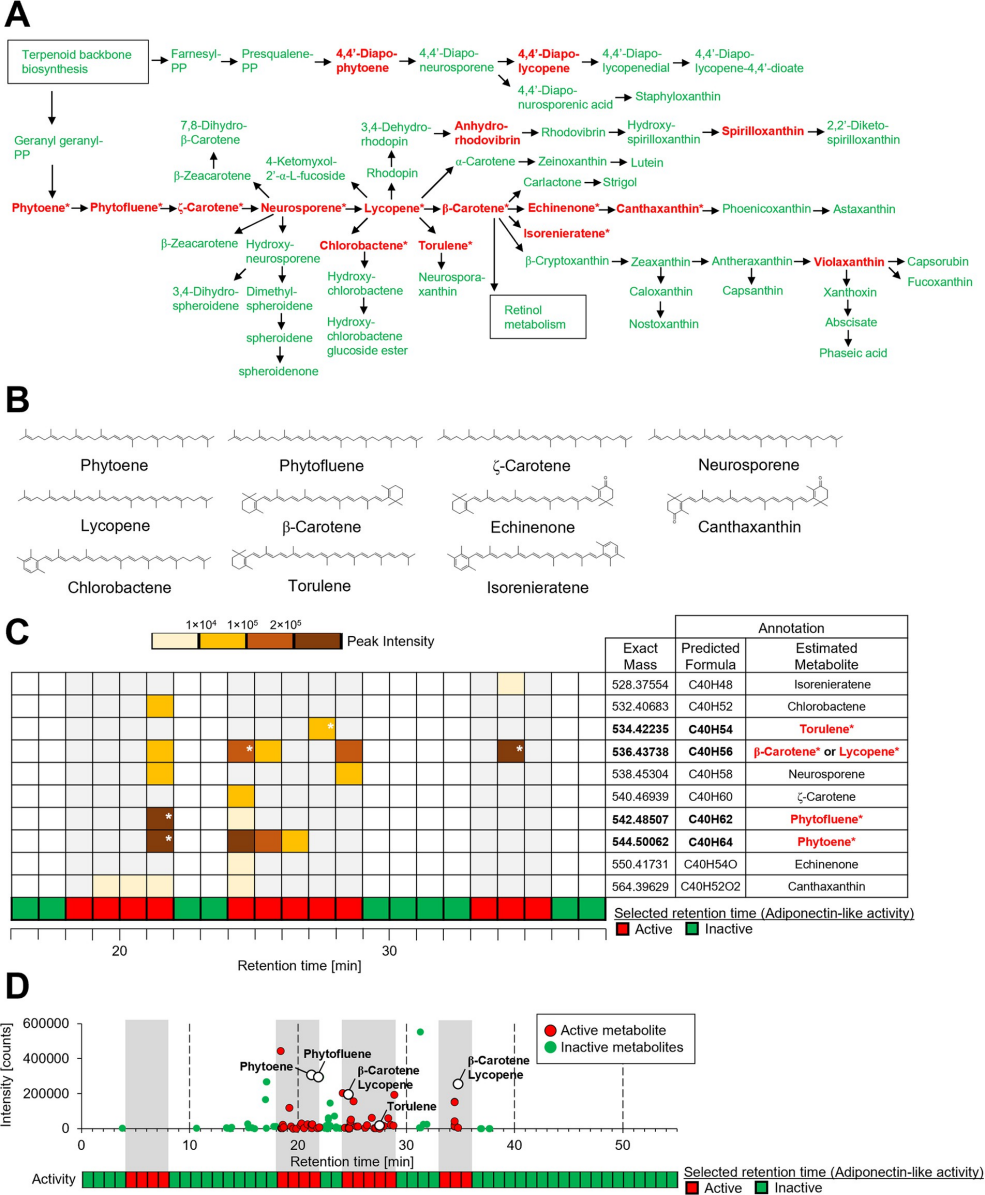
Profiling of screened tomato carotenoids in terms of their metabolic pathway and chemical nature. **(A)** The biosynthesis pathway of carotenoids based on the KEGG database. The carotenoids denoted in red font are metabolites annotated in tomato extract by screening. Carotenoids denoted by asterisks were selected for further analysis. **(B)** Structures of the carotenoids chosen for detailed screening based on the biosynthesis pathway of carotenoids. **(C)** Chemical properties of the selected carotenoids for further analysis. Left: Heatmap of the peak intensity of carotenoids in the tomato metabolome. The peak intensity of the selected carotenoids is denoted by asterisks. Right: The exact mass, predicted molecular formula and estimated metabolite corresponding to each peak on the heatmap on the left. Bottom: Heatmap of the retention time range with adiponectin-like activity. Red, active ranges; green, inactive ranges. **(D)** The active carotenoids screened for further analysis. Top: Plot of the intensity and retention time of the tomato carotenoids. Grey zones denote retention times exhibiting adiponectin-like activity. Red dots denote active metabolites. Green dots denote inactive metabolites. White dots with compound names indicate carotenoids selected for further analysis.

In the next screening step, a detailed analysis of the 60 active carotenoids selected by integrating the biological assay and the non-targeted metabolite analysis was performed in terms of their metabolic pathways and chemical nature. The 60 active carotenoids were mapped to the Kyoto Encyclopedia of Genes and Genomes (KEGG) database, and 16 carotenoids were registered in the KEGG carotenoid biosynthesis pathway (Fig 5A, denoted in red). Interestingly, the screened carotenoids were highly clustered in the carotenoid biosynthesis pathway (between phytoene and canthaxanthin, besides chlorobactene, torulene and isorenieratene). Thus, 11 carotenoids were chosen from this pathway (denoted in red and by asterisks in Fig 5A and 5B) and their peak intensities in the retention time range that selected in terms of adiponectin-like activity were examined (Fig 5C). From 18–22 min retention time, phytoene and phytofluene exhibited high peak intensities; from 33–36 min, β-carotene or lycopene displayed high peak intensities. Therefore, we focused on phytoene, phytofluene and β-carotene/lycopene. From 24–29 min retention time, β-carotene/lycopene displayed high peak intensity. In addition, in the same retention time range, torulene, neurosporene, and ζ-carotene exhibited relatively higher peak intensities. We hence selected torulene, as a representative of a compound with a distinct chemical structure. In sum, five carotenoids were chosen, namely phytoene, phytofluene, β-carotene, lycopene and torulene (denoted by asterisks in Fig 5C and 5D).

**Fig 6:**
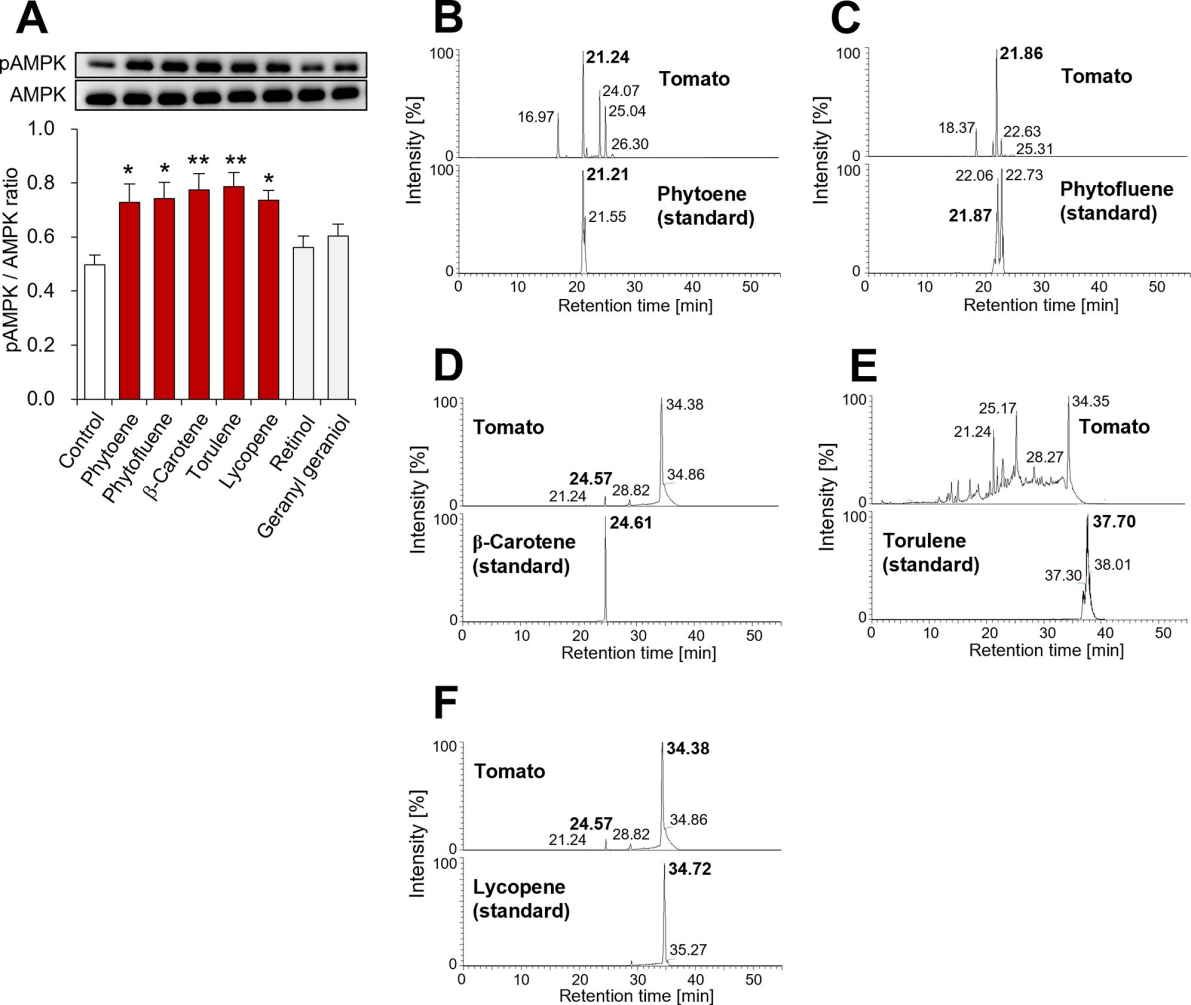
Identification of metabolite that activate adiponectin signaling pathway in tomato. **(A)** Effect of screened carotenoids on AMPK phosphorylation in C2C12 myotubes. Geranylgeraniol and retinol were examined as a precursor and degradation product of carotenoids, respectively. C2C12 myotubes were incubated with each carotenoid (1 μM), geranylgeraniol (1 μM) or retinol (1 μM) for 10 min. Total cell protein was extracted from the treated C2C12 myotubes and analyzed by western blotting. **(B)–(F)** Extracted ion chromatograms of **(B)** phytoene (m/*z* = 545.508), **(C)** phytofluene (m/*z* = 543.492), **(D)** β-carotene (m/*z* = 537.445), **(E)** torulene (m/*z* = 535.429) and **(F)** lycopene (m/*z* = 537.445). Data are presented as mean ± SEM from independent experiments (n = 6/group). *p < 0.05, **p < 0.01 vs. control. pAMPK, phosphorylated AMPK; AMPK, total AMPK.

To identify the compounds with adiponectin-like activity in tomato, authentic samples of the five selected carotenoids were evaluated in their biological activity and chemical properties. The five carotenoids enhanced AMPK phosphorylation, while the carotenoid precursor and degradation product, geranylgeraniol and retinol, did not significantly affect AMPK phosphorylation (Fig 6A). To identify the active metabolites based on their chemical properties by mass spectrometry, the retention times of authentic samples of the five selected carotenoids were evaluated. The elution times of authentic samples of phytoene, phytofluene, β-carotene and lycopene agreed well with the retention times of the putative molecules in the tomato metabolome (Fig 6B–6D and 6F). However, the elution time of authentic torulene was inconsistent with that of the corresponding putative molecule in the metabolome (Fig 6E). These observations suggested that four of the tomato-derived carotenoids (phytoene, phytofluene, β-carotene and lycopene) were metabolites that activate adiponectin signaling pathway.

### Evaluation of the effects of identified carotenoids on the adiponectin signaling pathway

**Fig 7:**
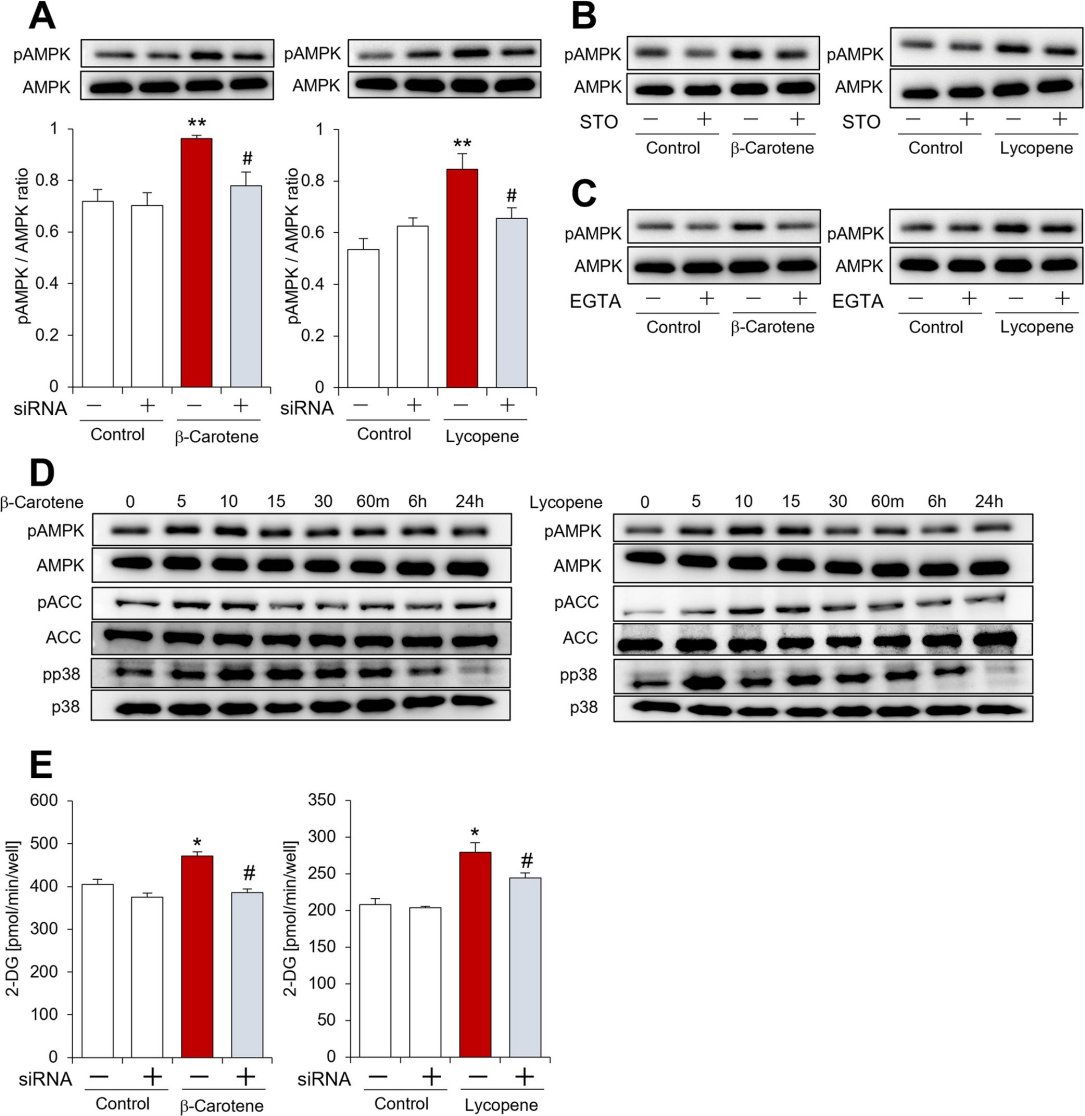
Evaluation of the effect of identified carotenoids on the adiponectin signaling pathway. **(A)**–**(C)** Effect of β-carotene or lycopene on AMPK phosphorylation in C2C12 myotubes in the presence and absence of **(A)** AdipoR siRNA, **(B)** ST-609 and **(C)** EGTA. C2C12 myotubes were pre-incubated for 6 h with STO-609 (1 μg/mL) or for 20 min with EGTA (5 mM), and subsequently treated for 10 min with β-carotene (1 μM) or lycopene (1 μM). The total cell protein was extracted from C2C12 myotubes and analyzed by western blotting. **(D)** Effect of β-carotene or lycopene on AMPK, ACC, and p38 phosphorylation in C2C12 myotubes. The C2C12 myotubes were incubated for the indicated times with β-carotene (1 μM) or lycopene (1 μM). **(E)** Effect of β-carotene or lycopene on the glucose uptake of C2C12 myotubes in the presence and absence of AdipoR siRNA. The C2C12 myotubes were incubated with β-carotene (1 μM) or lycopene (1 μM) for 10 min. See experimental details in the Methods section. Data are presented as mean ± SEM from independent experiments (n = 4–6/group). *p < 0.05, **p < 0.01 vs. control. ^**#**^*p* < 0.05 vs. carotenoid alone. pAMPK, phosphorylated AMPK; AMPK, total AMPK; pACC, phosphorylated ACC; ACC, total ACC; pp38, phosphorylated p38; p38, total p38; STO, STO-609.

For the detailed biological analysis of the potency of the identified carotenoids on the previously described adiponectin signaling pathway [7,34], β-carotene and lycopene were further examined as they are the major carotenoids of food. The treatment of C2C12 myotubes with β-carotene or lycopene significantly induced the phosphorylation of AMPK, whereas this effect was abrogated by AdipoR siRNA (Fig 7A). In addition, Ca^2+^/calmodulin-dependent protein kinase kinase (CaMKK) inhibition by the selective inhibitor STO-609 suppressed AMPK phosphorylation stimulated by β-carotene or lycopene (Fig 7B). EGTA, a Ca^2+^ chelator, also decreased the phosphorylation of AMPK induced by β-carotene or lycopene (Fig 7C). These data demonstrated that AdipoR, CaMKK and Ca^2+^ influx are required for the AMPK phosphorylation stimulated by β-carotene and lycopene, similar to adiponectin. Furthermore, in agreement with adiponectin signaling, β-carotene and lycopene increased the level of acetyl-CoA carboxylase (ACC), p38 and AMPK phosphorylation at the tested times. The ACC and AMPK phosphorylation induced by β-carotene or lycopene peaked at 5–15 min, while p38 phosphorylation persisted for 1–6 h (Fig 7D). Similar trends for ACC, AMPK and p38 phosphorylation associated with AdipoR have been reported [35,36]. In addition, β-carotene and lycopene promoted glucose uptake in C2C12 myotubes, which was subsequently inhibited by AdipoR siRNA treatment (Fig 7E). These findings indicated that β-carotene and lycopene activate adiponectin signaling pathway, including AMPK phosphorylation.

## Discussion

In this study, we identified β-carotene and lycopene as food-derived metabolites that activate adiponectin signaling pathway with AMPK phosphorylation by integration of biological assay and non-targeted metabolite analysis. Extract obtained from mature tomato phosphorylated AMPK in C2C12 myotubes and abrogation of the effect in AdipoR-knock down cells, suggesting that tomato contains metabolites with adiponectin-like activity. The non-targeted metabolite analysis of tomato revealed the presence of 721 metabolites that included carotenoids, lipids, and flavonoids among others. By integrating these 721 metabolites and the biological assay of HPLC fractions with the AMPK phosphorylation based on their retention times of liquid chromatography, we performed a screen for metabolites with adiponectin-like activity from tomato. This activity-based metabolite profiling revealed the presence of diverse 273 metabolites including carotenoids that could potentially activate adiponectin signaling pathway. We chose β-carotene and lycopene, the major carotenoids of food, for further investigation of their adiponectin-like activity. Both metabolites activated the previous reported signaling pathway of adiponectin.

The present findings demonstrate that β-carotene and lycopene activate adiponectin signaling pathway, and thus this novel property of these major carotenoids may contribute to the amelioration of glucose metabolism disorder through dietary intake of tomato. In previous reports, the intake of tomato has been closely associated with a reduced risk of a variety of chronic diseases, including glucose metabolism disorders [11–13]. It is commonly assumed that the beneficial effects of tomato intake are linked to the major carotenoids, β-carotene and lycopene [37,38]. It has been reported that the intake of carotenoids and plasma β-carotene concentration are inversely correlated with fasting plasma glucose levels and insulin resistance [39,40]. Plasma glucose and fasting insulin levels decrease with the increase of serum lycopene levels [41]. To date, the effects of these carotenoids have been mainly attributed to their anti-oxidant and anti-inflammatory properties [42–44]. Our results might suggest a new mechanism by which intake of tomato ameliorates glucose metabolism disorder.

To activate adiponectin signaling pathway with AMPK phosphorylation, carotenoids should be hydrophobic and not extensively modified structurally. In this study, we showed that tomato-derived carotenoids induced AMPK phosphorylation, similar to an adiponectin. However, the structure of carotenoid that is necessary for adiponectin-like activity is unclear. Carotenoids are C_40_ isoprenoid polyene compounds that are present as colourful pigments in various constituents of the human diet, including vegetables, fruits, seaweeds, and other natural products. Carotenoids are highly structurally diverse, which contributes to their widespread biological activity [45,46]. Therefore, the AMPK phosphorylation activity of major dietary carotenoids was determined to explore their structure-activity relationships (S2A Fig). As a consequence, α-carotene and fucoxanthin elicited AMPK phosphorylation, whereas lutein, zeaxanthin, capsanthin, β-cryptoxanthin, astaxanthin, geranylgeraniol (the carotenoid precursor) and retinol (the degradation product of carotenoid) did not significantly affect AMPK phosphorylation in C2C12 myotubes (S2B Fig). These results suggested that the carotenoids upstream of the biosynthesis pathway (up to β-carotene, torulene and α-carotene) tend to enhance the phosphorylation of AMPK (S2C Fig), namely hydrophobic and not extensively chemically modified (e.g., oxidized or hydroxylated) structure is required for carotenoids to activate adiponectin signaling pathway. Consistent with this prediction, the tomato carotenoids screened in our study possessed these chemical properties (denoted in red in Fig 5A and 5B). On the other hand, fucoxanthin, which harbours epoxy, hydroxyl, and allene groups, also significantly increased the phosphorylation of AMPK (S2A and S2B Fig). This result contrasted with the data for the other carotenoids examined in the present study, while agreeing with a previous report that fucoxanthin elicits phosphorylation of AMPK in mouse hepatocyte FL83B cells [47]. To further clarify the structure-activity relationship of carotenoids activating adiponectin signaling pathway, further experiments are needed with carotenoids having a wide variety of chemical structures.

*Cis*-isomers of β-carotene and lycopene are favoured as carotenoids with adiponectin-like activity. β-carotene and lycopene have a molecular formula of C_40_H_56_ and possess long-chain conjugated double bonds. Although most of the β-carotene and lycopene in tomato and other foods are present in the all-*trans* configuration, various kinds of *cis*-isomers of these carotenoids are naturally present [48]. Several reports demonstrated that biological functions of carotenoid are changed between their *cis* and *trans* isomers [48–50]. Therefore, we examined whether the adiponectin-like activity of the identified carotenoids is altered in the *cis* and *trans* isomers. In an animal model of genetic diabetes (*db*/*db* mice), in which the hypoglycemic effects of adiponectin and AdipoRon have been reported [8], oral administration of *cis*-isomerized lycopene was shown to lower plasma glucose levels more strongly than all-*trans* lycopene (S3A Fig). The AMPK phosphorylation that is induced by the naturally occurring and major *cis*-isomers of these carotenoids (9-*cis* or 13-*cis*) [51,52] were significantly higher than those of the corresponding all-*trans* configurations in C2C12 myotubes (S3B and S3C Fig). This effect was consistent with its hypoglycemic effect in mice. These results suggested that *cis*-isomers of β-carotene and lycopene are favoured to activate adiponectin signaling pathway. Additionally, in case of isomers of lycopene, only AMPK phosphorylation elicited by the 13-*cis* isomer was significantly higher than that of the corresponding all-*trans* configuration (S3C Fig). These observations suggested that the *cis* configuration centered on conjugated double bonds of carotenoids might be beneficial for their adiponectin-like activities. To further clarify this structure-activity relationship of *cis*-*trans* isomers of carotenoid, further experiments are needed with carotenoids having diverse *cis* configurations.

Non-carotenoids that activate adiponectin signaling pathway might be present in tomato. In this study, tomato extract that included a wide variety of metabolites displayed adiponectin-like activity in the phosphorylation of AMPK (Fig 1). By integrating the biological assay and non-targeted metabolite analysis, we revealed the presence of 273 diverse metabolites that could potentially activate adiponectin signaling pathway from 721 metabolites of tomato (Figs 3 and 4). Here, we focused on carotenoids that were highly valued in integrated screening processes. However, non-carotenoids that activate adiponectin signaling pathway, including AMPK phosphorylation might be present in these 273 metabolites. In fact, carotenoids were not detected in the metabolites eluted at the selected 4–8 min retention time, which caused the increase of AdipoR-dependent phosphorylation of AMPK in C2C12 myotubes (Fig 4H). Some of the metabolites eluted during the 4–8 min retention time were reported to induce the phosphorylation of AMPK. Hispidulin, annotated in Fig 4B, is a naturally occurring flavonoid [53]. It stimulates AMPK phosphorylation in SKOV3 human ovarian cancer cells [54], in SMMC7721 and Bel7402 hepatocellular carcinoma cells [55], and in GBC-SD human gallbladder carcinoma cells [56]. Nootkatone, annotated in Fig 4E, is a naturally occurring sesquiterpene and ketone [57]. Nootkatone was reported to increase the phosphorylation of AMPK in Hepa1-6 mouse hepatoma cells and C2C12 myotubes [58], in A549 human lung adenocarcinoma cells [59]. On the other hand, the concentration and incubation time of hispidulin and nootkatone that produce significant increases of AMPK phosphorylation in previous reports differ from those of carotenoids and the AdipoRon, synthetic adiponectin mimetic [8]. In addition, annotation in metabolomics is presumptive and requires identification using authentic standards. In fact, the identification of the five selected carotenoids, the retention times of each of the four species were consistent, but the elution times of torulene were not (Fig 6E). It is unclear whether these annotated metabolites of tomato, except for carotenoids, activate adiponectin signaling pathway. Further experiments using authentic chemical standards are needed to identify non-carotenoids that activate adiponectin signaling pathway from 273 metabolites in tomato.

The precise molecular mechanism of the action of identified carotenoids in activating the adiponectin signaling pathway is not fully understood. By integrating 721 tomato-derived metabolites and the activity of HPLC fractions on AdipoR-dependent phosphorylation of AMPK, we screened for metabolites that activate adiponectin signaling pathway from tomato. Whereas this activity-based metabolite profiling efficiently identified β-carotene and lycopene as active metabolites with adiponectin-like activity, the precise molecular mechanism of these carotenoids remains unclear. Recently, the crystal structure of human AdipoR was determined at high resolution, which represents a novel class of receptor structure [60]. A synthetic small-molecule (AdipoRon) and several peptides can mimic adiponectin actions through agonistic binding to the AdipoR, suggesting that small-molecule has the potential to function as an AdipoR agonist [8–10]. Therefore, one possibility is that β-carotene and lycopene may function as agonists of AdipoR in activating adiponectin signaling pathway. On the other hand, whereas the agonist-receptor interaction is generally predicted to be strongly dependent on the chemical structure of the agonist, the identified carotenoids do not share structural features with AdipoRon and the peptides described above. Further studies, including in silico analyses, are needed to investigate the agonistic action of the identified carotenoids. In the present study, we showed that the identified carotenoids activate various elements of the adiponectin signaling pathway related to phosphorylation (Fig 7). It has also been reported that adiponectin signaling involves the translocation of proteins such as glucose transporter type 4 (Glut4) and liver kinase B1 (LKB1) [61,62]. In skeletal muscle cells, adiponectin increases glucose uptake via translocation of Glut4 to the plasma membrane in a downstream signal of AMPK phosphorylation. Similar to adiponectin, the identified carotenoids promoted Glut4 translocation to the plasma membrane (S4A Fig) and enhanced glucose uptake (Fig 7E). Glut4 translocation by other carotenoid in skeletal muscle has also been reported [63]. Like CaMKK, LKB1 is another upstream kinase of AMPK. LKB1 induces adiponectin-stimulated phosphorylation of AMPK by translocation from the nucleus to the cytoplasm in muscle cells. However, the identified carotenoids did not induce the above-mentioned LKB1 translocation (S4B Fig), suggesting that the identified carotenoids may not completely mimic the action of adiponectin. Further investigations are needed to fully elucidate the precise molecular mechanism of how β-carotene and lycopene activate adiponectin signaling pathway.

In conclusion, integration of biological assay and non-targeted metabolite analysis allowed the screening for metabolites that activate adiponectin signaling pathway from tomato. The screening suggested that four carotenoids have adiponectin-like activity. We further analyzed β-carotene and lycopene, the tomato major carotenoids. Both induced AMPK phosphorylation through AdipoR, CaMKK, and Ca^2+^ influx. These carotenoids activated glucose uptake via AdipoR in C2C12 myotubes. All the observed activities are characteristic of adiponectin. The collective findings demonstrate that the food-derived carotenoids, β-carotene and lycopene, activate the adiponectin signaling pathway, including AMPK phosphorylation.

## Supporting information

S1 Fig AdipoR-knockdown of C2C12 myotubes. **(A)** AdipoR-knockdown of C2C12 myotubes by AdipoR1-specific siRNA transfection. The total cell protein was extracted from treated C2C12 myotubes and analyzed by western blotting. Data are presented as mean ± SEM from independent experiments (n = 6 /group). **p < 0.01 vs. control.
(TIF)

S2 Fig Structure-activity relationship of carotenoids and AMPK phosphorylation. **(A)** Structure of the major carotenoids found in common dietary sources. **(B)** Effect of major carotenoids found in dietary sources on AMPK phosphorylation in C2C12 myotubes. C2C12 myotubes were incubated with each carotenoid (1 μM), geranylgeraniol (1 μM) or retinol (1 μM) for 10 min. The total cell protein was extracted from treated C2C12 myotubes and analyzed by western blotting. **(C)** The carotenoid biosynthesis pathway based on the KEGG database. Metabolites denoted by red font elicit AMPK phosphorylation. Metabolites denoted by green font do not elicit AMPK phosphorylation. Data are presented as mean ± SEM from independent experiments (n = 6/group). **p < 0.01 vs. control. pAMPK, phosphorylated AMPK; AMPK, total AMPK.
(TIF)

S3 Fig Structure-activity relationship of β-carotene and lycopene stereoisomers and AMPK phosphorylation. **(A)** Plasma glucose levels after oral administration of all-*trans* lycopene (200mg/kg body weight) or *cis*-lycopene (200mg/kg body weight) in 8-week-old male *db*/*db* mice. The isomerized lycopene was used as *cis*-lycopene in animal experiment. (For further details of the preparation of isomerized lycopene, see Materials and methods) The composition of isomerized lycopene is as follows (12.4% 13*cis*, 11.9% 9*cis*, 17.6% 5*cis*, and 20.6% other *cis*-lycopene). Data are presented as mean ± SEM (n = 6–7/group). *p < 0.05 vs. control. **(B)** Structures of the naturally occurring and major *cis*-isomers of β-carotene and lycopene. **(C)** Effect of β-carotene and lycopene *cis*-isomers on AMPK phosphorylation in C2C12 myotubes. C2C12 myotubes were incubated with each carotenoid (1 μM) for 10 min. The total cell protein was extracted from treated C2C12 myotubes and analyzed by western blotting. Data are presented as mean ± SEM from independent experiments (n = 5–7/group). *p < 0.05 vs. control. ^**#**^*p* < 0.05 vs. all-*trans* carotenoid. pAMPK, phosphorylated AMPK; AMPK, total AMPK.
(TIF)

S4 Fig Translocation of Glut4 and LKB1 associated with the adiponectin signaling pathway **(A)** Effect of β-carotene and lycopene on Glut4 translocation to the plasma membrane in C2C12 myotubes. C2C12 myotubes were incubated with each carotenoid (5 μM) for 1 hr. The plasma membrane fraction was extracted from treated C2C12 myotubes and analyzed by western blotting. **(B)** Effect of β-carotene and lycopene on LKB1 translocation to the cytoplasm in C2C12 myotubes. C2C12 myotubes were incubated with each carotenoid (5 μM) for 10 min. The cytoplasm fraction was extracted from treated C2C12 myotubes and analyzed by western blotting. Glut4 PM, Glut4 protein in plasma membrane fraction; Glut4 CL, Glut4 protein in cell lysate; LKB1 CY, LKB1 protein in cytoplasm fraction; LKB1 CL, LKB1 protein in cell lysate.
(TIF)

S5 Fig Full-length western blots of Figures. (PDF)

S1 Table The detailed metabolome of hydrophobic extract of tomato. (XLSX)

S1 Raw images (PDF)
