# Supplementary figures and images for: Integration of bioassay and non-target metabolite analysis of tomato reveals that β-carotene and lycopene activate the adiponectin signaling pathway, including AMPK phosphorylation
Source: PLoS One. 2022 Jul 1;17(7):e0267248. doi: 10.1371/journal.pone.0267248 (PMC9249195; doi:10.1371/journal.pone.0267248)

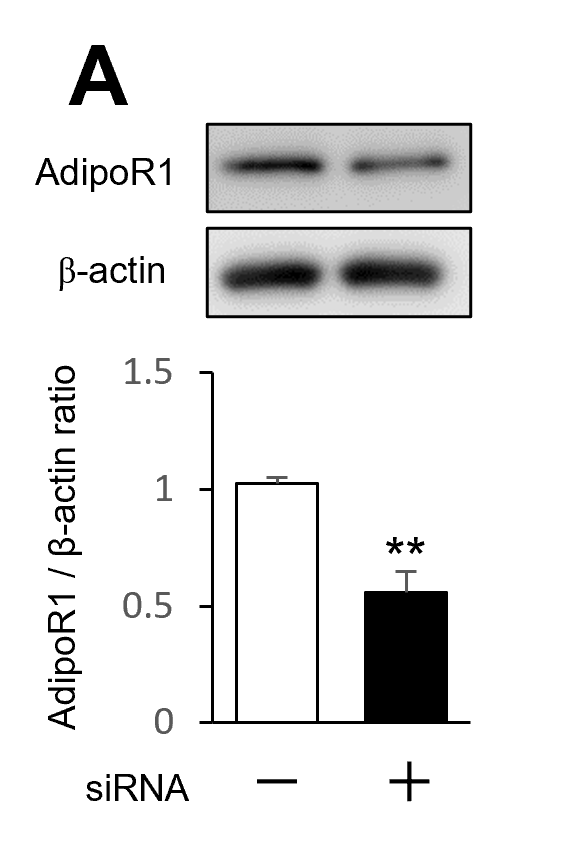

Supplement: S1 Fig — (A) AdipoR-knockdown of C2C12 myotubes by AdipoR1-specific siRNA transfection. The total cell protein was extracted from treated C2C12 myotubes and analyzed by western blotting. Data are presented as mean ± SEM from independent experiments (n = 6 /group). **p < 0.01 vs. control. (TIF) [file pone.0267248.s001.tif]

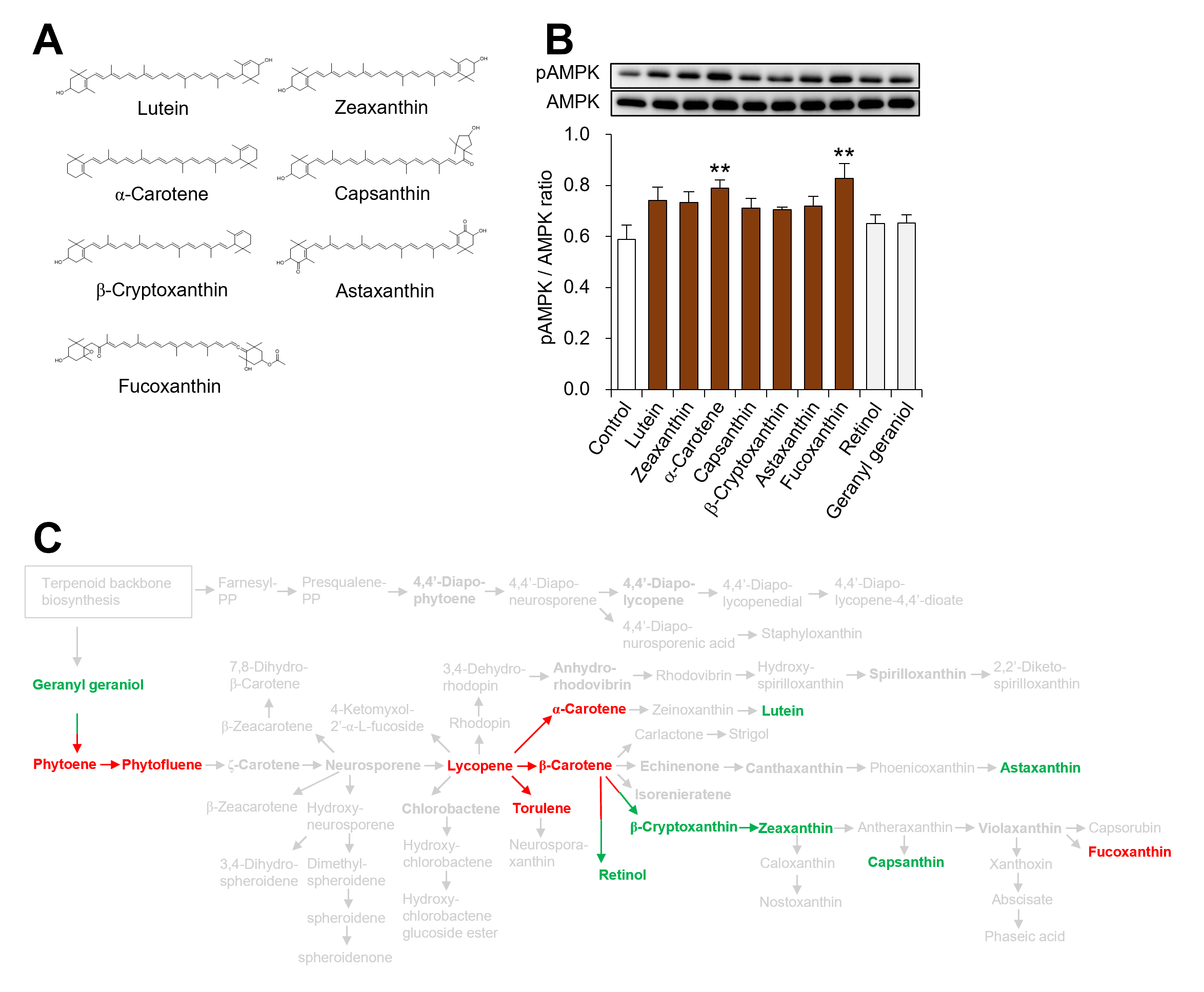

Supplement: S2 Fig — (A) Structure of the major carotenoids found in common dietary sources. (B) Effect of major carotenoids found in dietary sources on AMPK phosphorylation in C2C12 myotubes. C2C12 myotubes were incubated with each carotenoid (1 μM), geranylgeraniol (1 μM) or retinol (1 μM) for 10 min. The total cell protein was extracted from treated C2C12 myotubes and analyzed by western blotting. (C) The carotenoid biosynthesis pathway based on the KEGG database. Metabolites denoted by red font elicit AMPK phosphorylation. Metabolites denoted by green font do not elicit AMPK phosphorylation. Data are presented as mean ± SEM from independent experiments (n = 6/group). **p < 0.01 vs. control. pAMPK, phosphorylated AMPK; AMPK, total AMPK. (TIF) [file pone.0267248.s002.tif]

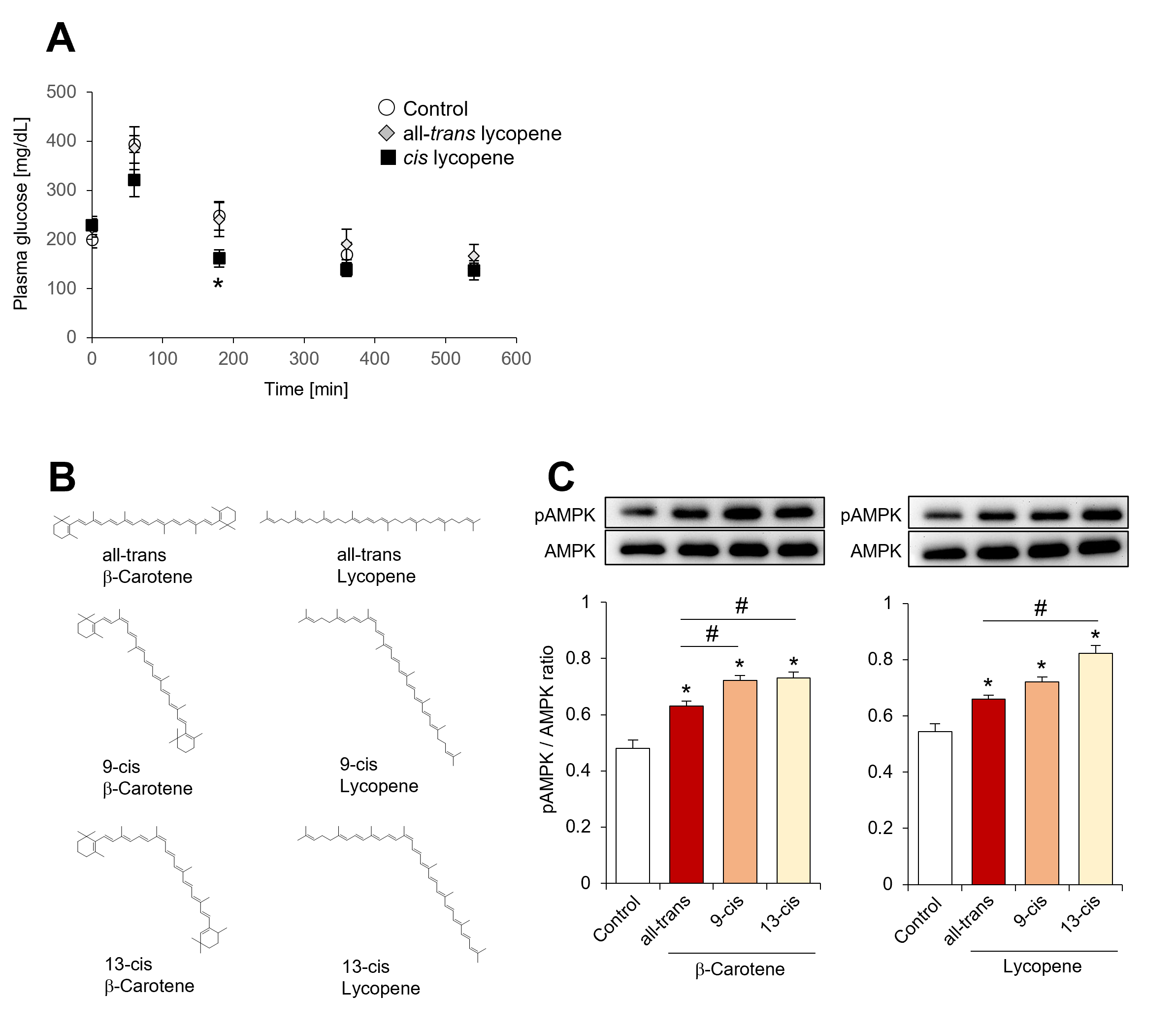

Supplement: S3 Fig — (A) Plasma glucose levels after oral administration of all-trans lycopene (200mg/kg body weight) or cis-lycopene (200mg/kg body weight) in 8-week-old male db/db mice. The isomerized lycopene was used as cis-lycopene in animal experiment. (For further details of the preparation of isomerized lycopene, see Materials and methods) The composition of isomerized lycopene is as follows (12.4% 13cis, 11.9% 9cis, 17.6% 5cis, and 20.6% other cis-lycopene). Data are presented as mean ± SEM (n = 6–7/group). *p < 0.05 vs. control. (B) Structures of the naturally occurring and major cis-isomers of β-carotene and lycopene. (C) Effect of β-carotene and lycopene cis-isomers on AMPK phosphorylation in C2C12 myotubes. C2C12 myotubes were incubated with each carotenoid (1 μM) for 10 min. The total cell protein was extracted from treated C2C12 myotubes and analyzed by western blotting. Data are presented as mean ± SEM from independent experiments (n = 5–7/group). *p < 0.05 vs. control. #p < 0.05 vs. all-trans carotenoid. pAMPK, phosphorylated AMPK; AMPK, total AMPK. (TIF) [file pone.0267248.s003.tif]

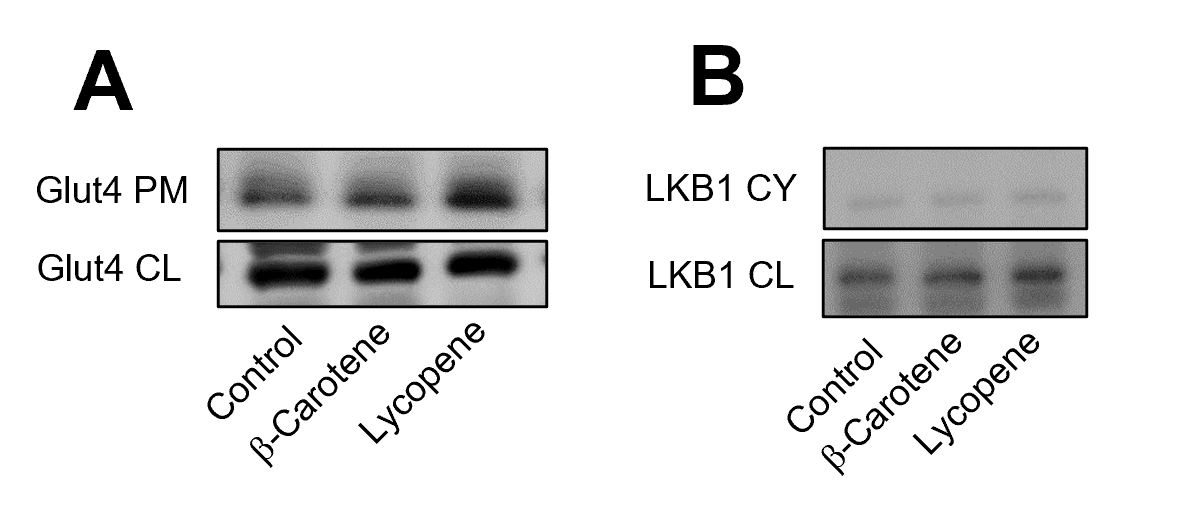

Supplement: S4 Fig — Translocation of Glut4 and LKB1 associated with the adiponectin signaling pathway (A) Effect of β-carotene and lycopene on Glut4 translocation to the plasma membrane in C2C12 myotubes. C2C12 myotubes were incubated with each carotenoid (5 μM) for 1 hr. The plasma membrane fraction was extracted from treated C2C12 myotubes and analyzed by western blotting. (B) Effect of β-carotene and lycopene on LKB1 translocation to the cytoplasm in C2C12 myotubes. C2C12 myotubes were incubated with each carotenoid (5 μM) for 10 min. The cytoplasm fraction was extracted from treated C2C12 myotubes and analyzed by western blotting. Glut4 PM, Glut4 protein in plasma membrane fraction; Glut4 CL, Glut4 protein in cell lysate; LKB1 CY, LKB1 protein in cytoplasm fraction; LKB1 CL, LKB1 protein in cell lysate. (TIF) [file pone.0267248.s004.tif]
