# Supplementary material for: Integration of bioassay and non-target metabolite analysis of tomato reveals that β-carotene and lycopene activate the adiponectin signaling pathway, including AMPK phosphorylation
Source: PLoS One. 2022 Jul 1;17(7):e0267248. doi: 10.1371/journal.pone.0267248 (PMC9249195; doi:10.1371/journal.pone.0267248)
Supplement: S1 Raw images — (PDF) [file pone.0267248.s007.pdf]

Supporting information Figure 5. (Full-length Western blots)

Full-length Western blots of Figure 1A-C.

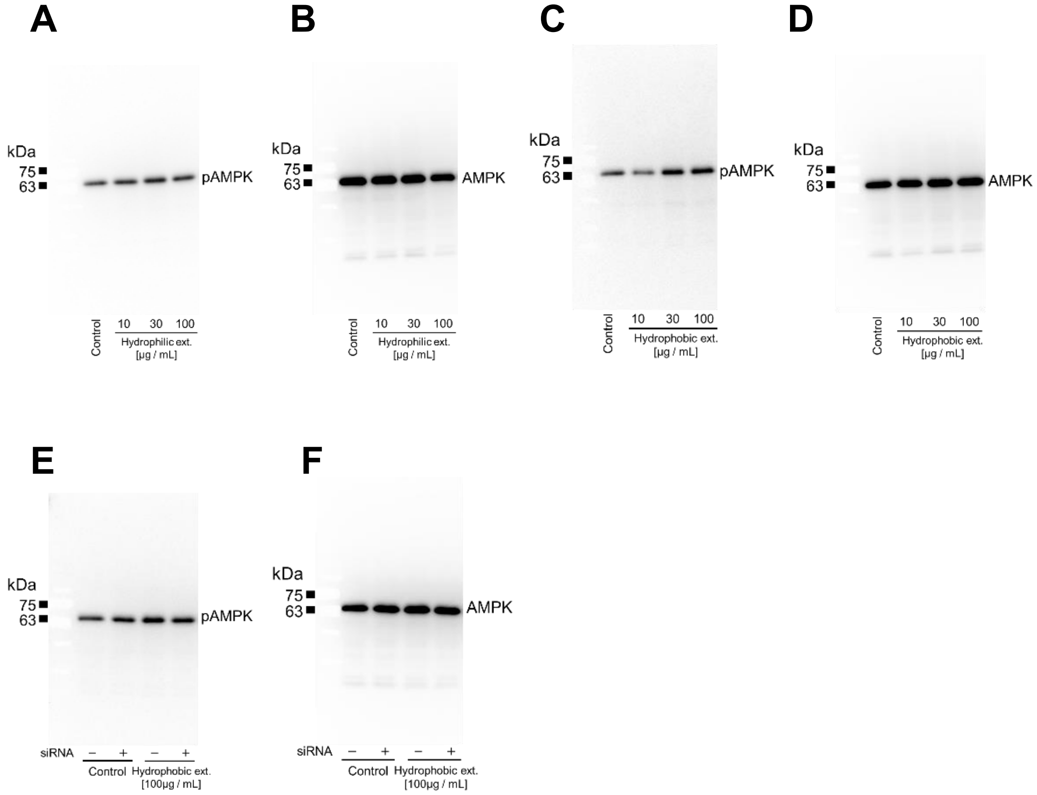

Full-length western blots of Figure 1 A–C. (A)–(B) Effect of the hydrophilic tomato extract on **(A)** AMPK phosphorylation, **(B)** total AMPK in C2C12 myotubes. **(C)–(D)** Effect of the hydrophobic tomato extract on **(C)** AMPK phosphorylation, **(D)** total AMPK in C2C12 myotubes. **(E)–(F)** Effect of the hydrophobic tomato extract on **(E)** AMPK phosphorylation, **(F)** total AMPK in the presence and absence of AdipoR siRNA. pAMPK, phosphorylated AMPK; AMPK, total AMPK.

Full-length Western blots of Figure 2A.

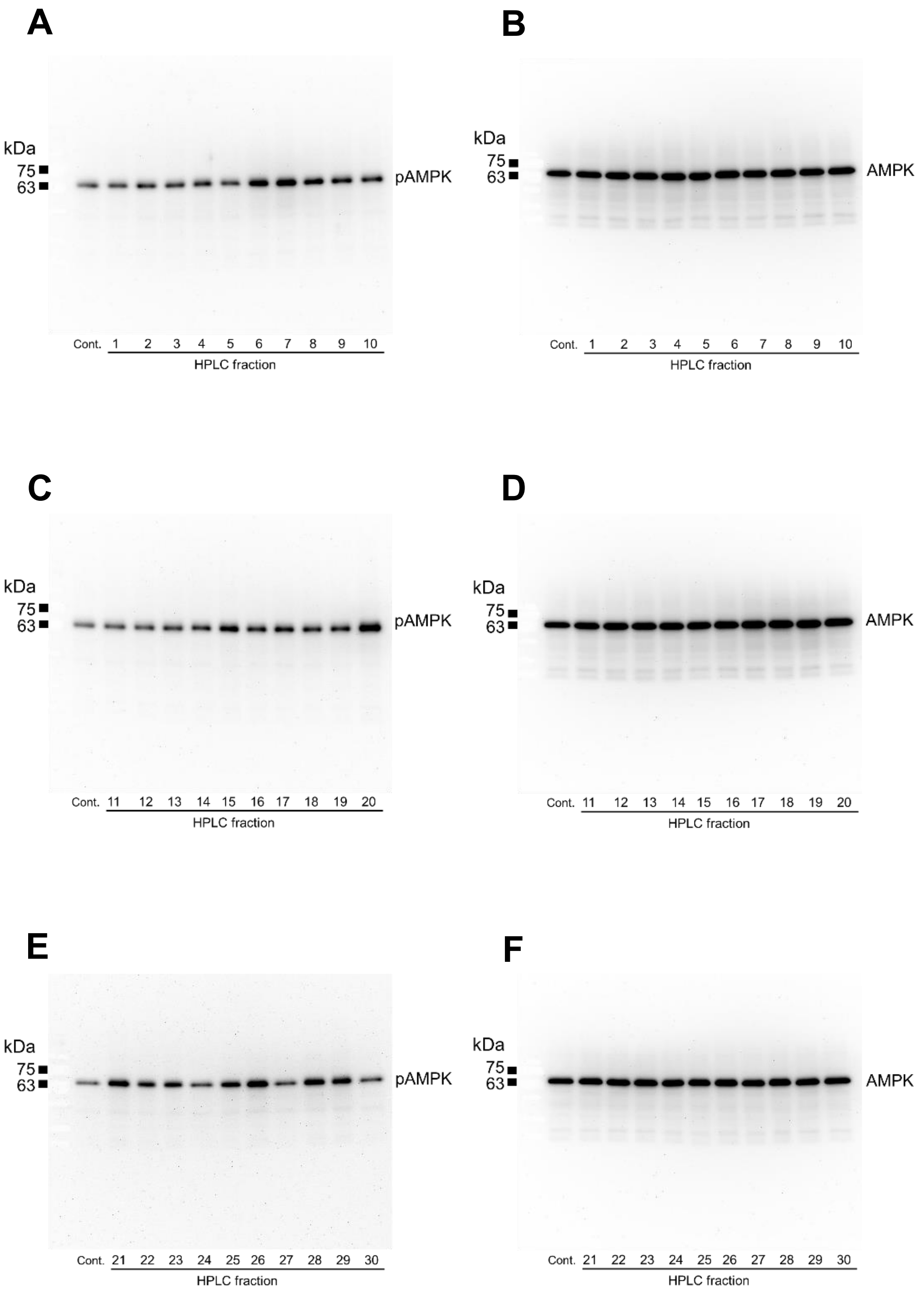

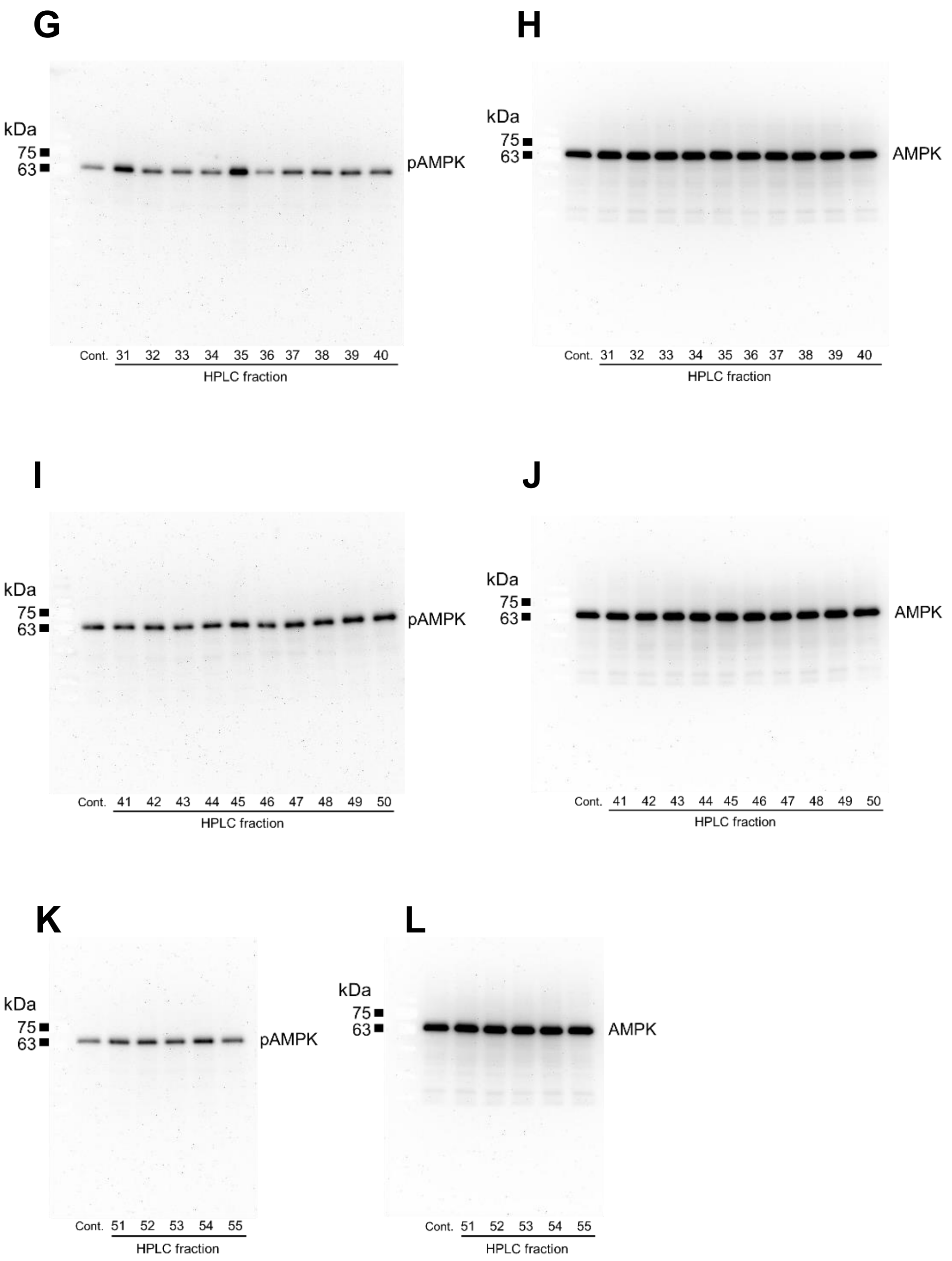
G
kDa
75
63
pAMPK
Cont. 31 32 33 34 35 36 37 38 39 40
HPLC fraction
H
kDa
75
63
AMPK
Cont. 31 32 33 34 35 36 37 38 39 40
HPLC fraction
I
kDa
75
63
pAMPK
Cont. 41 42 43 44 45 46 47 48 49 50
HPLC fraction
J
kDa
75
63
AMPK
Cont. 41 42 43 44 45 46 47 48 49 50
HPLC fraction
K
kDa
75
63
pAMPK
Cont. 51 52 53 54 55
HPLC fraction
L
kDa
75
63
AMPK
Cont. 51 52 53 54 55
HPLC fraction

Full-length western blots of Figure 2A. **(A)–(B)** Effect of the HPLC fraction HPLC fractions no.1–10 on **(A)** AMPK phosphorylation, **(B)** total AMPK in C2C12 myotubes. **(C)–(D)** Effect of the HPLC fractions no.11–20 on **(C)** AMPK phosphorylation, **(D)** total AMPK in C2C12 myotubes. **(E)–(F)** Effect of the HPLC fractions no.21–30 on **(E)** AMPK phosphorylation, **(F)** total AMPK in C2C12 myotubes. **(G)–(H)** Effect of the HPLC fractions no.31–40 on **(G)** AMPK phosphorylation, **(H)** total AMPK in C2C12 myotubes. **(I)–(J)** Effect of the HPLC fractions no.41–50 on **(I)** AMPK phosphorylation, **(J)** total AMPK in C2C12 myotubes. **(K)–(L)** Effect of the HPLC fractions no.51–55 on **(K)** AMPK phosphorylation, **(L)** total AMPK in C2C12 myotubes. pAMPK, phosphorylated AMPK; AMPK, total AMPK.

Full-length Western blots of Figure 2B.

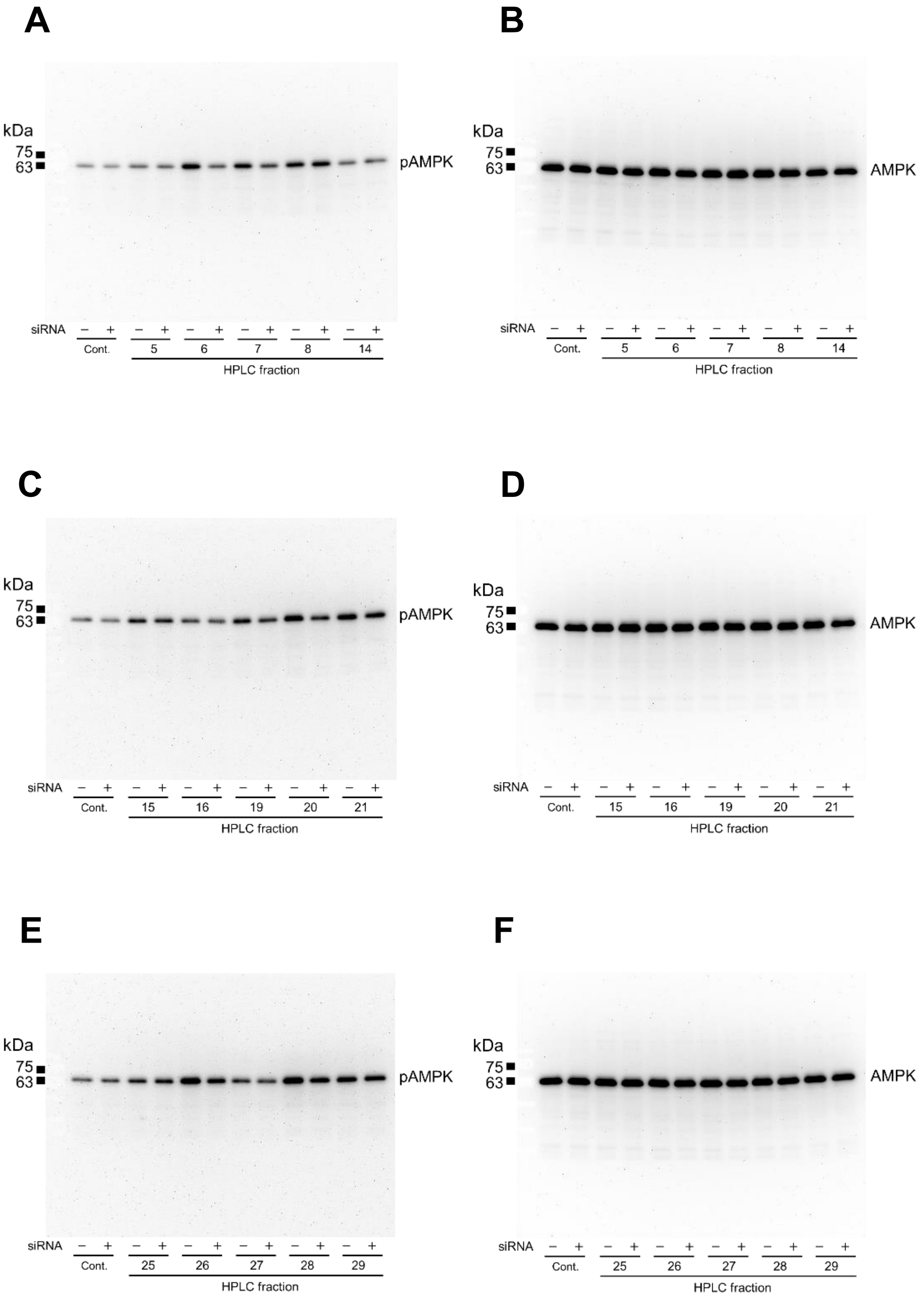

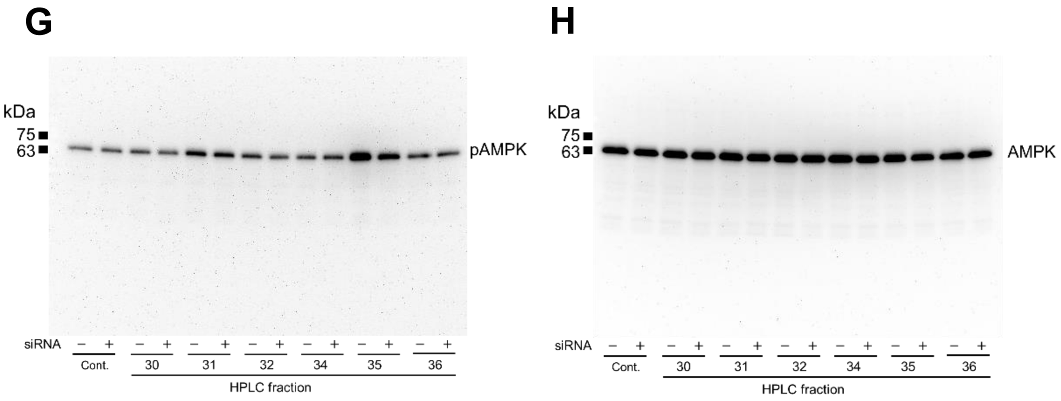

Full-length western blots of Figure 2B. (A)–(B) Effect of the HPLC fraction HPLC fractions no.5, 6, 7, 8, and 14 on **(A)** AMPK phosphorylation, **(B)** total AMPK in C2C12 myotubes in the absence and presence of AdipoR siRNA. **(C)–(D)** Effect of the HPLC fractions no.15, 16, 19, 20, and 21 on **(C)** AMPK phosphorylation, **(D)** total AMPK in C2C12 myotubes in the absence and presence of AdipoR siRNA. **(E)–(F)** Effect of the HPLC fractions no.25, 26, 27, 28, and 29 on **(E)** AMPK phosphorylation, **(F)** total AMPK in C2C12 myotubes in the absence and presence of AdipoR siRNA. **(G)–(H)** Effect of the HPLC fractions no.30, 31, 32, 34, 35, and 36 on **(G)** AMPK phosphorylation, **(H)** total AMPK in C2C12 myotubes in the absence and presence of AdipoR siRNA. pAMPK, phosphorylated AMPK; AMPK, total AMPK.

Full-length Western blots of Figure 6A.

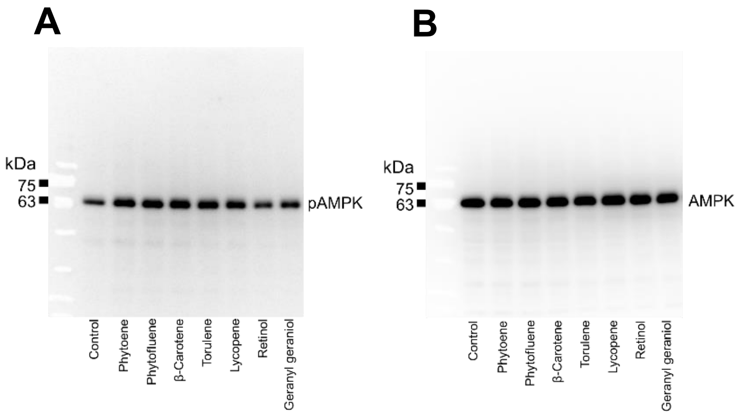

Full-length western blots of Figure 6A. (A)–(B) Effect of screened carotenoids on **(A)** AMPK phosphorylation, **(B)** total AMPK in C2C12 myotubes. pAMPK, phosphorylated AMPK; AMPK, total AMPK.

Full-length Western blots of Figure 7A-D.

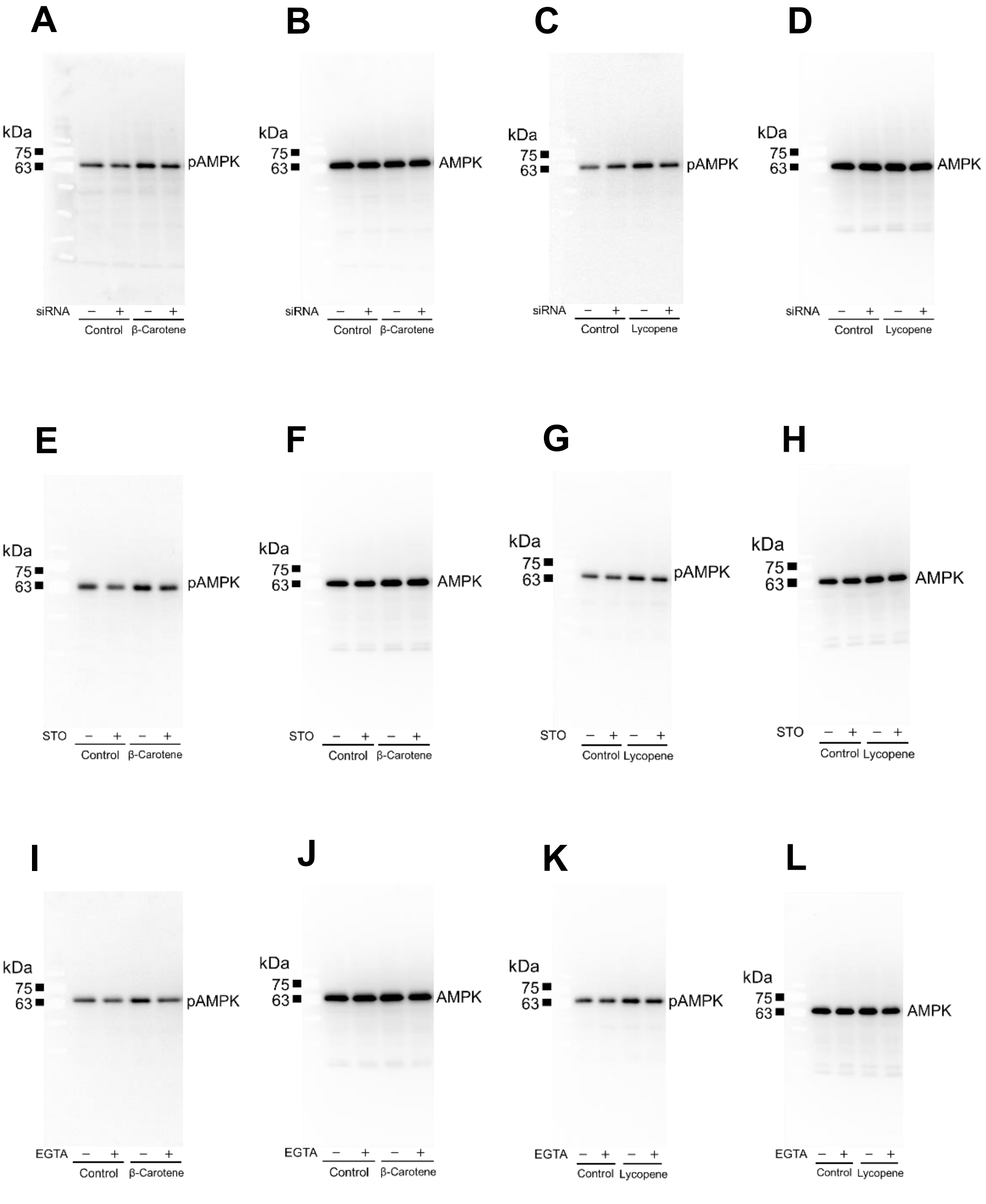

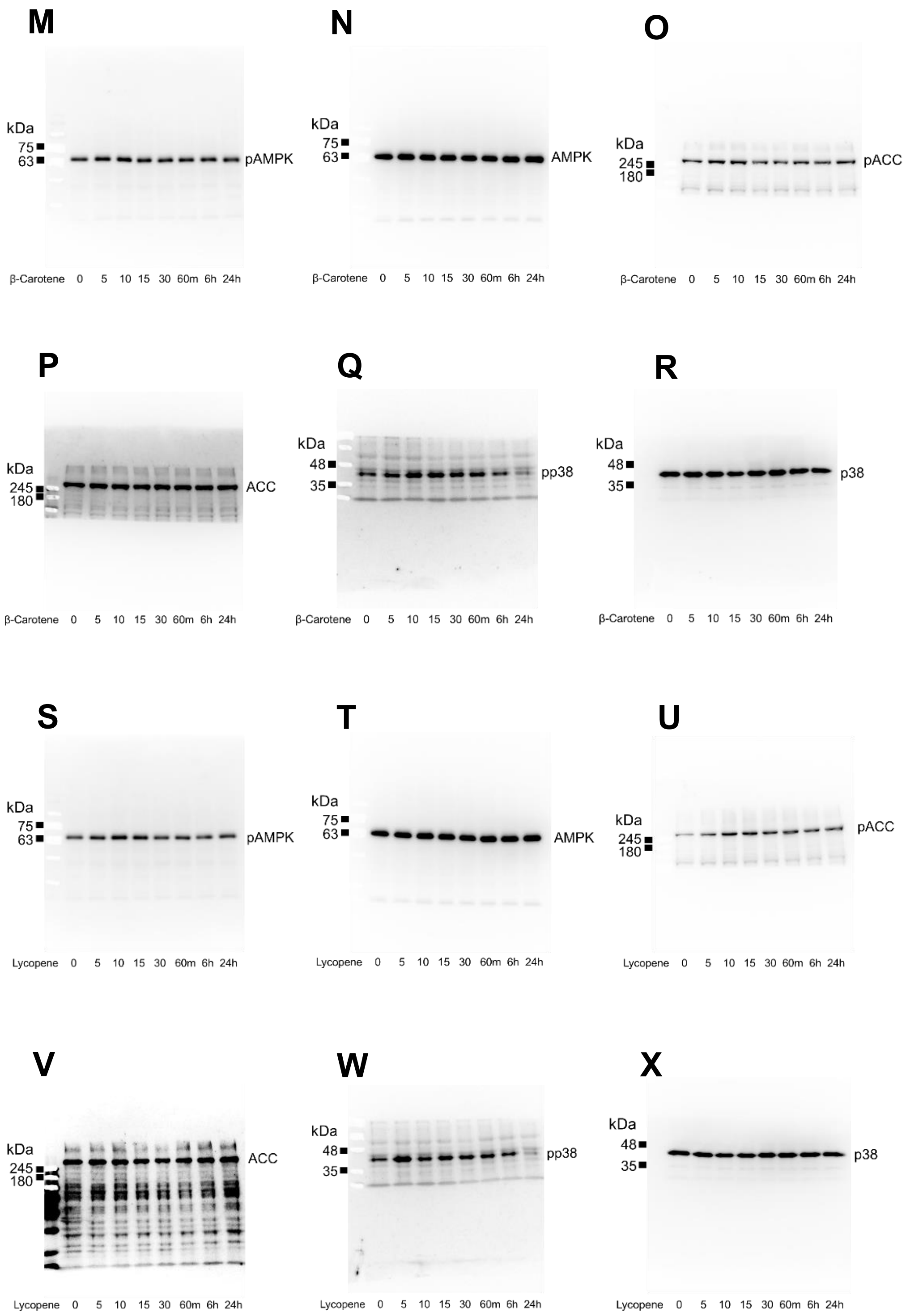
M
kDa
75
63
pAMPK
β-Carotene 0 5 10 15 30 60m 6h 24h
N
kDa
75
63
AMPK
β-Carotene 0 5 10 15 30 60m 6h 24h
O
kDa
245
180
pACC
β-Carotene 0 5 10 15 30 60m 6h 24h
P
kDa
245
180
ACC
β-Carotene 0 5 10 15 30 60m 6h 24h
Q
kDa
48
35
pp38
β-Carotene 0 5 10 15 30 60m 6h 24h
R
kDa
48
35
p38
β-Carotene 0 5 10 15 30 60m 6h 24h
S
kDa
75
63
pAMPK
Lycopene 0 5 10 15 30 60m 6h 24h
T
kDa
75
63
AMPK
Lycopene 0 5 10 15 30 60m 6h 24h
U
kDa
245
180
pACC
Lycopene 0 5 10 15 30 60m 6h 24h
V
kDa
245
180
ACC
Lycopene 0 5 10 15 30 60m 6h 24h
W
kDa
48
35
pp38
Lycopene 0 5 10 15 30 60m 6h 24h
X
kDa
48
35
p38
Lycopene 0 5 10 15 30 60m 6h 24h

Full-length western blots of Figure 7A–D. (A)–(B) Effect of β-carotene on **(A)** AMPK phosphorylation, **(B)** total AMPK in C2C12 myotubes in the presence and absence of AdipoR siRNA. **(C)–(D)** Effect of lycopene on **(C)** AMPK phosphorylation, **(D)** total AMPK in C2C12 myotubes in the presence and absence of AdipoR siRNA. **(E)–(F)** Effect of β-carotene on **(E)** AMPK phosphorylation, **(F)** total AMPK in C2C12 myotubes in the presence and absence of STO-609. **(G)–(H)** Effect of lycopene on **(G)** AMPK phosphorylation, **(H)** total AMPK in C2C12 myotubes in the presence and absence of STO-609. **(I)–(J)** Effect of β-carotene on **(I)** AMPK phosphorylation, **(J)** total AMPK in C2C12 myotubes in the presence and absence of EGTA. **(K)–(L)** Effect of lycopene on **(K)** AMPK phosphorylation, **(L)** total AMPK in C2C12 myotubes in the presence and absence of EGTA. **(M)–(N)** Effect of β-carotene on **(M)** AMPK phosphorylation, **(N)** total AMPK in C2C12 myotubes at the tested times. **(O)–(P)** Effect of β-carotene on **(O)** ACC phosphorylation, **(P)** total ACC in C2C12 myotubes at the tested times. **(Q)–(R)** Effect of β-carotene on **(Q)** p38 phosphorylation, **(R)** total p38 in C2C12 myotubes at the tested times. **(S)–(T)** Effect of lycopene on **(S)** AMPK phosphorylation, **(T)** total AMPK in C2C12 myotubes at the tested times. **(U)–(V)** Effect of lycopene on **(U)** ACC phosphorylation, **(V)** total ACC in C2C12 myotubes at the tested times. **(W)–(X)** Effect of lycopene on **(W)** p38 phosphorylation, **(X)** total p38 in C2C12 myotubes at the tested times. pAMPK, phosphorylated AMPK; AMPK, total AMPK; pACC, phosphorylated ACC; ACC, total ACC; pp38, phosphorylated p38; p38, total p38.

Full-length Western blots of Supplementary information Figure 1A.

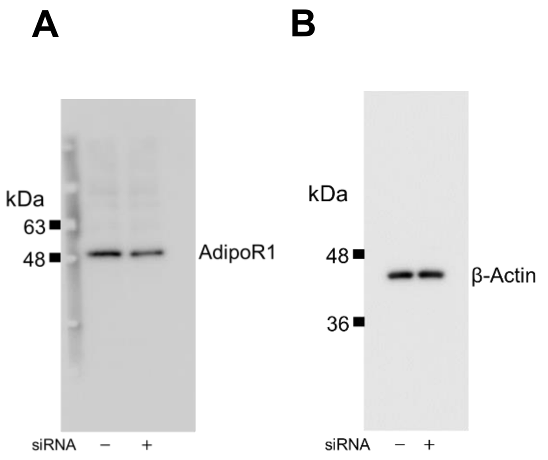

Full-length western blots of Supplementary information Figure 1A. (A)–(B) AdipoR-knockdown of C2C12 myotubes on **(A)** AdipoR1, **(B)** β-actin in the presence and absence of AdipoR siRNA. AdipoR1, adiponectin receptor 1.

Full-length Western blots of Supplementary information Figure 2B.

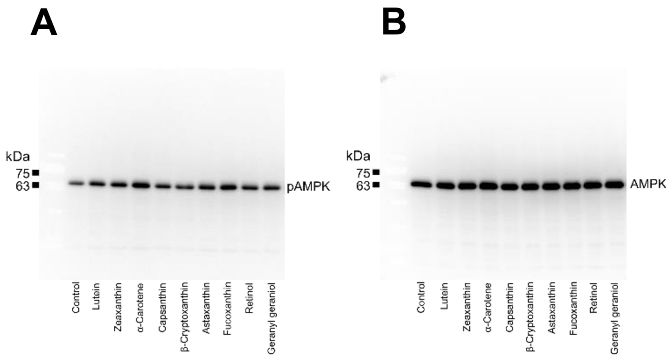

Full-length western blots of Supplementary information Figure 2B. (A)–(B) Effect of major carotenoids found in dietary sources on **(A)** AMPK phosphorylation, **(B)** total AMPK in C2C12 myotubes. pAMPK, phosphorylated AMPK; AMPK, total AMPK.

Full-length Western blots of Supplementary information Figure 3C.

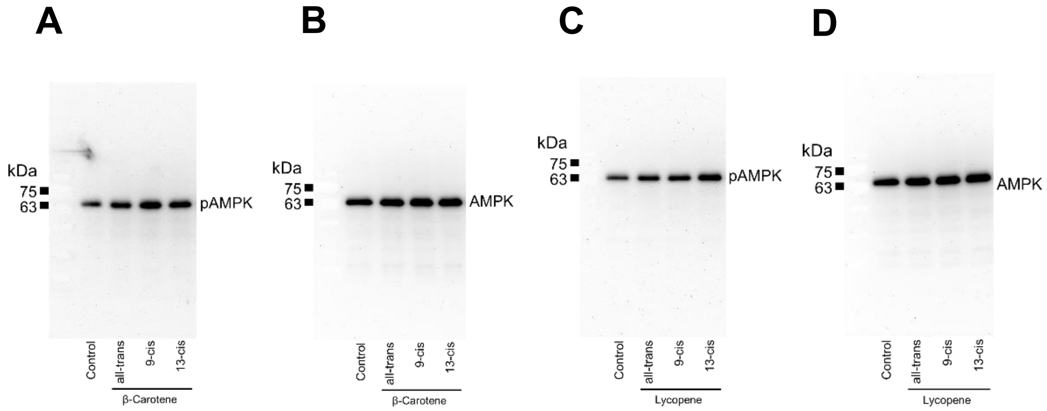

Full-length western blots of Supplementary information Figure 3C. (A)–(B) Effect of β-carotene stereoisomers on **(A)** AMPK phosphorylation, **(B)** total AMPK in C2C12 myotubes. **(C)–(D)** Effect of lycopene stereoisomers on **(C)** AMPK phosphorylation, **(D)** total AMPK in C2C12 myotubes. pAMPK, phosphorylated AMPK; AMPK, total AMPK.

Full-length Western blots of Supplementary information Figure 4A-B.

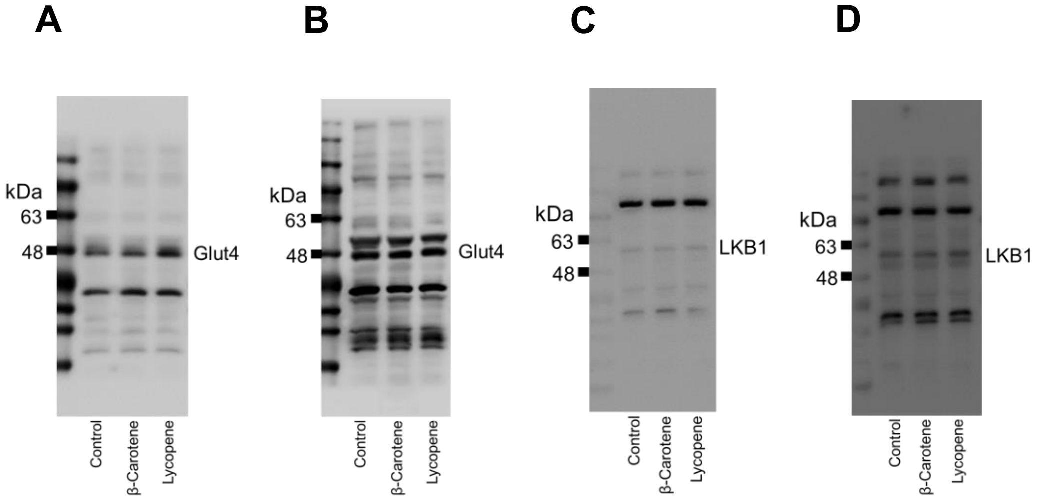

Full-length western blots of Supplementary information Figure 4A–B. (A)–(B) Effect of β-carotene and lycopene on Glut4 translocation **(A)** plasma membrane fraction, **(B)** cell lysate in C2C12 myotubes. **(C)–(D)** Effect of β-carotene and lycopene on LKB1 translocation **(C)** cytoplasm fraction, **(D)** cell lysate in C2C12 myotubes.
